# Maternal perinatal depression and infant self-regulation: A meta-analytic review

**DOI:** 10.1017/S0954579425100837

**Published:** 2025-11-21

**Authors:** Emily R. Padrutt, Daniel Berry, Ellie Schwartzman, Sylia Wilson

**Affiliations:** Institute of Child Development, University of Minnesota, Minneapolis, MN, USA

**Keywords:** Depression, infancy, meta-analysis, perinatal, self-regulation

## Abstract

Infant self-regulation is shaped by early physiological systems and caregiver-infant co-regulatory interactions. Maternal perinatal (pre- and/or postnatal) depression may affect these processes and infants’ development of this critical construct. However, literature addressing the association between maternal perinatal depression and infant self-regulation has been mixed. We conducted a pre-registered meta-analysis of the association between maternal perinatal depression and several self-regulation constructs (e.g., effortful control, executive function) measured during the first 2 years of life. We included 68 reports comprising 193 effect sizes and 16,722 mother-infant dyads. On average, studies included an equal number of male and female infants, and, for most (68%) studies, most participants were White. Average infant age ranged from 0 – 16 months. Three-level random effects meta-analytic models indicated a small, significant overall association, with higher levels of depression associated with lower self-regulation (*r* = −.10, 95% CI = −.14, −.06, *p* < .001). There was substantial heterogeneity in this pooled effect. Subsequent analyses indicated moderation by methodological and conceptual variables. Evidence that maternal perinatal depression is associated with lower infant self-regulation underscores the importance of supporting dyads experiencing perinatal depression. Clarifying this association highlights a critical next step of examining potential causal processes linking maternal and infant well-being.

## Introduction

Self-regulation is a critical capacity first evident in infancy and shaped by early experiences, which may include exposure to maternal depression. In this meta-analysis, we first review the construct of self-regulation and infants’ development of self-regulation and then overview theoretical and empirical evidence indicating maternal perinatal (pre- and postnatal) depression may be a particularly salient exposure affecting early self-regulation. We then present a meta-analytic review of the empirical literature examining associations between maternal perinatal depression and self-regulation (broadly construed) across the first 2 years of life. We close by considering the strengths and limitations of the literature, placing our findings in the context of existing conceptual models, and considering implications for research and policy or clinical work.

### Self-regulation

#### Conceptualization of self-regulation

Self-regulation abilities predict many domains of adaptive functioning (Robson et al., 2020), including academic and cognitive performance (Best et al., 2011), emotional health (Aldao et al., 2016), and relational functioning (Fitzsimons & Finkel, 2011; Ringoot et al., 2021). There is notable variation in current conceptualizations of self-regulation (e.g., Blair et al., 2012; Nigg, 2017; Vink et al., 2020). Some inconsistency in the conceptualization of this construct stems from its parallel emergence across sub-disciplines with overlapping but different perspectives on the construct, including the temperament literature, more cognitively focused areas of the literature, and more affectively focused areas (Erdmann & Hertel, 2019). Ongoing debates about the construct include its overlap with co-regulation or external regulatory processes (Eisenberg et al., 2010), whether it is conceptualized as a stable trait or an in-the-moment process (Cole et al., 2019; Diaz & Eisenberg, 2015), and the separability of reactivity and regulation (Adrian et al., 2011). Overall, based on a review of prominent models (Blair & Ku, 2022; Nigg, 2017), we conceptualize self-regulation broadly as a domain-general umbrella term that includes interacting bottom-up/reactive/automatic and top-down/deliberate/control processes that are internally initiated^1^ and regulate physiology, cognition, emotion, and behavior. Self-regulation is thought to develop hierarchically, with later-developing, more complex and volitional components (e.g., executive function) building on earlier-developing, more basic and automatic components (e.g., physiological regulation). In line with this framework, we consider the broad construct of self-regulation to encompass constructs specific to the regulation of physiology (e.g., autonomic regulation, adrenocortical regulation), cognition (e.g., executive function, attention control), emotion (e.g., emotion regulation), and behavior (e.g., behavior regulation). We also include constructs described within the temperament literature (e.g., effortful control, behavioral inhibition).

While much of the self-regulation literature, and some previous reviews (e.g., Power et al., 2021), have focused on specific constructs within the self-regulation domain, we aimed to consider the self-regulation construct more broadly for three reasons. First, the broad construct of self-regulation is implicated frequently in the field of developmental psychopathology as a core aspect of healthy socioemotional development, and challenges in self-regulation are central to theories of the development of psychopathology (Caspi & Moffitt, 2018; Goodman & Gotlib, 1999). Second, conceptualizations of constructs underlying self-regulation (e.g., effortful control) vary substantially across the literature (Nigg, 2017). Given this variation, parsing the self-regulation literature by specific construct does not yield a uniform construct.^2^ Finally, taking a broad approach in our conceptualization of self-regulation allowed us to consider various more meaningful characteristics of the self-regulation construct (e.g., independent regulation versus co-regulation, trait-like or process-oriented conceptualizations) as moderators.

#### Self-regulation development

The development of self-regulation begins prenatally and continues throughout infancy, childhood, and adolescence (Cerritelli et al., 2021; Feldman, 2009; Geva & Feldman, 2008; Kopp, 1982; Panagiotakopoulos & Neigh, 2014). The earliest forms of physiological regulation, as indexed, for example, by fetal heart rate variability (Kinsella & Monk, 2009), are supported by autonomic nervous system (ANS) and hypothalamic-pituitary-adrenal (HPA) axis development and can be detected prenatally with physiological assessments (Cerritelli et al., 2021; Panagiotakopoulos & Neigh, 2014). During the first 2 to 3 months after birth, neurophysiological processes and reflexive behaviors, which can be reliably assessed with structured clinician assessments (Brazelton & Nugent, 2011), regulate infant physiological homeostasis and arousal states (Feldman, 2009; Kopp, 1982). Other early regulatory behaviors include rudimentary self-comforting, such as hand- or thumb-sucking, and early bids for caregiver regulatory support, such as distress vocalizations (Fox & Calkins, 2003). As these behaviors are readily observable, even from this early developmental stage, observation-based and caregiver-report assessments of self-regulation become possible. Later in the first year, infants begin to employ attentional and behavioral strategies to regulate emotions, such as averting their gaze from distressing stimuli and attempting to engage caregivers with positive vocalizations. Rudimentary inhibitory motor control also emerges around this time. In the second year of life, increases in and integration of attentional and inhibitory motor control capacities provide the foundation for various developmental competencies, including delaying gratification and beginning to comply with external demands (Fox & Calkins, 2003). In toddlerhood and beyond, prefrontal cortex development further facilitates attention regulation as a self-regulatory strategy that enables increasingly independent regulation of behavior, attention, and emotions (Feldman, 2009; Vink et al., 2020). Research supports the hierarchical nature of this development by demonstrating that difficulties in foundational domains (e.g., physiological regulation) contribute to difficulties in later-developing abilities (Feldman, 2009; Wu et al., 2021).

Critically, the early development of self-regulation occurs in the context of social relationships, primarily the caregiver-infant relationship (Beeghly & Tronick, 2011; Fox & Calkins, 2003; Vink et al., 2020). The Mutual Regulation Model emphasizes caregivers’ role in self-regulation development during infancy and suggests that self-regulation develops in the dyadic context, based on the contributions of an infant subsystem, a caregiver subsystem, and the dynamic interaction between them (Gianino & Tronick, 1988). Thus, understanding long-term self-regulation development requires examination of not only infant regulatory capacities, but also of caregiver contributions and contextual factors affecting them.

### Maternal depression and infant self-regulation

A substantial literature demonstrates that infants, children, and adolescents of mothers^3^ with depression show differences across domains of functioning, including difficulties with self-regulation (e.g., Babineau et al., 2015; Bates et al., 2020; Pinto et al., 2023), compared with those of mothers without depression (Gotlib et al., 2023). Additionally, there are theoretical reasons maternal depression may influence infant self-regulation development. Specifically, maternal physiological processes that accompany elevated prenatal depressive symptoms may affect the development of fetal/infant physiological regulation (Field et al., 2004; Galbally et al., 2020; Kinsella & Monk, 2009; Monk et al., 2011), which is foundational to broader infant self-regulation (Feldman, 2009). Additionally, maternal postnatal depressive symptoms may make it more difficult for mothers to engage in sensitive parenting behaviors that support the development of infant self-regulation (Bernard et al., 2018; Bernier et al., 2010). Thus, we expect that higher maternal perinatal depressive symptoms will be associated with lower infant self-regulation.

### The present meta-analysis

Maternal perinatal depression is remarkably prevalent, affecting up to one in five mother-infant dyads (Bauman et al., 2020), with prevalence likely even higher since the onset of the COVID-19 pandemic (Iyengar et al., 2021). Understanding the association between maternal depression and infant self-regulation is critical due to the hierarchical nature of self-regulation development; factors affecting self-regulation in infancy continue to shape self-regulation into later childhood and beyond and may serve as a vulnerability for the development of psychopathology or other difficulties (Goodman & Gotlib, 1999). However, the literature on this association has been mixed. Some results suggest higher levels of maternal perinatal depression are associated with lower infant self-regulation (e.g., Bates et al., 2020), some suggest no association (Conradt et al., 2013), and some suggest better infant self-regulation in the context of maternal depression (e.g., Khoury et al., 2016). These discrepant findings may be due to various factors: imprecision related to sampling error in individual studies with modest sample sizes, important demographic and methodological characteristics of studies that contribute to systematic differences in findings, and variation in conceptualizations of self-regulation across studies.

While previous reviews have examined associations between maternal anxiety or stress and offspring self-regulation (Korja et al., 2017); maternal depression and broader child socioemotional development, temperament, psychopathology, or behavior problems (Beck, 1998, 1999; Connell & Goodman, 2002; Goodman et al., 2011; Harris & Santos, 2020; Madigan et al., 2018; Martucci et al., 2021; Parsons et al., 2012; Petzoldt, 2018; Takegata et al., 2021; Waxler et al., 2011); maternal depression and child executive function (Power et al., 2021); and maternal depression and infant response to the Still Face Paradigm (Graham et al., 2018), to our knowledge, no previous reviews have examined the association between maternal perinatal depression and infant self-regulation.

In this pre-registered meta-analysis, we systematically reviewed and quantitatively synthesized the empirical literature examining the association between maternal depression and infant self-regulation. Despite evidence of mixed findings in the literature, given theoretical links between maternal perinatal depression and infant self-regulation, we hypothesized that more symptoms of maternal depression or a depression diagnosis would be associated with lower infant self-regulation. We also examined heterogeneity and potential demographic and methodological moderating variables. Our demographic moderators (infant sex and infant age at self-regulation assessment) were exploratory. We had directional hypotheses for some of our methodological moderators (sample type, type of depression score, and reliability of depression and self-regulation measures), for which we expected stronger associations in clinical samples, with continuous scores, and with more reliable measures. Other methodological moderators (method used to assess maternal depression and infant self-regulation, timing of maternal depression, and moderators related to self-regulation conceptualizations) were exploratory.

## Method

This study was pre-registered (https://osf.io/jw7t2/overview?view_only=76cb058fad0e42c9b584ec4c675ba0a1). Additional details, including deviations from preregistration, can be found below and in the Supplemental Methods.

### Inclusion and exclusion criteria

Inclusion criteria for the meta-analysis were (a) assessment of self-regulation or an underlying construct (see Table 1) among human infants ages 0 through 23 months, (b) assessment of maternal depression prior to or concurrently with assessment of infant self-regulation, and (c) availability of sufficient information for calculating effect size(s) of the association between maternal depression and infant self-regulation. We defined infancy as 0 – 23 months to encompass a wider range of the developmental change occurring in early self-regulation and to allow us to examine infant age as a moderator of our findings. We also included only studies assessing maternal depression prior to or concurrently^4^ with infant self-regulation to align with our broader theoretical model in which maternal depression influences infant self-regulation.

Exclusion criteria were (a) assessment of maternal depression after assessment of infant self-regulation, (b) intervention studies that did not provide associations prior to the intervention or separately for the control group, (c) case studies or studies with fewer than 10 participants, (d) studies not in English, and (e) studies with insufficient effect size information. We did not apply any exclusion criteria related to study population or study geographic location, and included studies came from across the world.

### Literature search

Our search and screening procedures were pre-registered, and deviations are noted. With the goals of capturing a comprehensive picture of early self-regulation and affording ourselves the ability to examine conceptual variations in the self-regulation construct as moderators, we conceptualized self-regulation as an umbrella term with many underlying constructs. We selected underlying constructs (Table 1) based on review of prominent models of self-regulation (Adrian et al., 2011; Barkley, 2001; Baumeister & Vohs, 2007; Beeghly et al., 2016; Blair & Ku, 2022; Diamond, 2013; Eisenberg & Spinrad, 2004; Eisenberg & Sulik, 2012; Feldman, 2009; Fox & Calkins, 2003; Hendry et al., 2016; Inzlicht et al., 2021; Kochanska et al., 2000; Kopp, 1982; Posner & Rothbart, 1998; Rothbart & Derryberry, 1981; Rothbart et al., 2003; Thompson, 1994; Zelazo & Carlson, 2020).

We searched four online databases (ERIC, Medline, PsycINFO, and ProQuest Dissertations). We searched titles, abstracts, and keywords of articles using a combination of search terms capturing the self-regulation-related constructs listed in Table 1 and depression (“depress*”) and targeting the infancy period (infan*, newborn*, neonat*, *baby*, or *babies*). We limited our search to studies in English and involving human subjects. To examine the past 10+ years of empirical literature, which have coincided with a substantial increase in interest in self-regulation, we searched for studies published between January 1, 2010 and November 30, 2023. Searches were conducted and records were exported on December 11, 2023.

The search produced 2,147 non-duplicate records (duplicates were identified manually by the first author). We first screened titles and abstracts of all records and excluded records that were not empirical, not in English, or did not include human subjects. We sought 1,036 records for full-text screening, of which five could not be retrieved. During full-text screening, we excluded 795 records that did not measure self-regulation or a related construct^5^ in the first 2 years after birth. We further excluded 99 records that did not measure maternal depression or measured it after measurement of infant self-regulation. We excluded four records reporting intervention studies that did not provide information on the association between maternal perinatal depression and infant self-regulation prior to the intervention (i.e., at baseline) or separately for the control group. Further, we excluded 61 records for which we were unable to estimate effect sizes for the association between maternal depression and infant self-regulation.^6^

**Figure 1.**
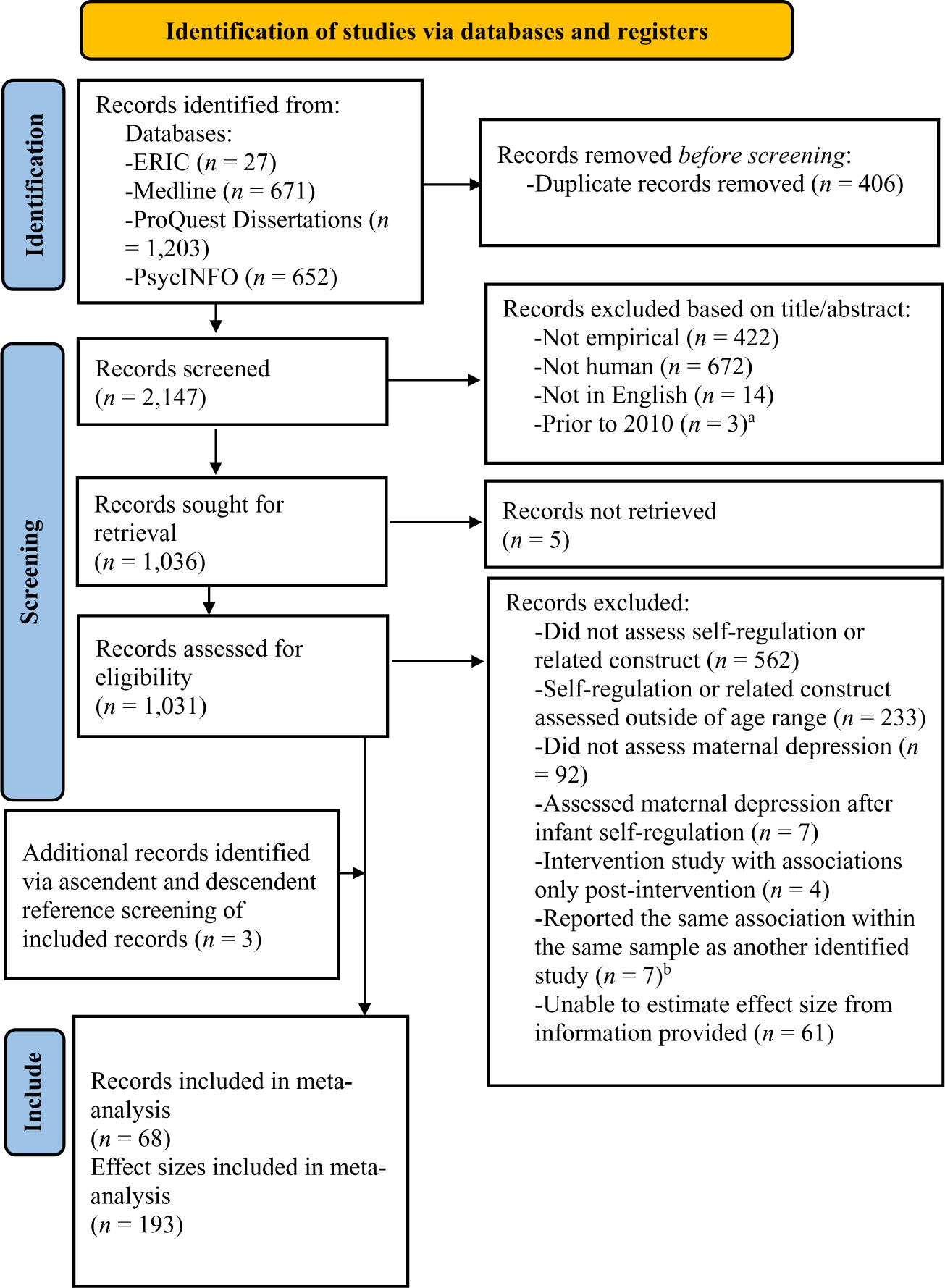
Flow diagram for literature search and screening procedure. *Note*. This figure is adapted from the preferred reporting items for systematic reviews and meta-analyses (PRISMA) flow diagram (Page et al., 2021). ^a^ The search was limited to the years 2010 – 2023. However, the years of three articles were indexed incorrectly in the databases and thus removed during title/abstract screening. ^b^ When we identified two or more records that presented the association of the same variables within the same (or a subset of the same) sample, we retained the record presenting the effect size with the largest *n*, or the most recent record if *ns* were identical. If the non-retained record(s) provided additional information not presented in the retained record about any of our coded variables, we retained this information for analyses.

We also examined reference lists of included records and records that cited included records for additional relevant records, which yielded three additional records that met inclusion criteria. Altogether, these search and screening procedures produced 68 reports that included 193 effect sizes and 16,722 unique mother-infant dyads that were included in the meta-analytic review. The search and screening procedure, which followed PRISMA guidelines (Page et al., 2021) is documented in Figure 1.

Twenty to twenty-five percent of records were second-screened to evaluate interrater reliability of screening criteria. Percent agreement was greater than 95% for each of the three screening steps, which were determining if 1) the record included an assessment of self-regulation or an underlying construct, 2) the self-regulation-related construct was assessed in the age range, and 3) the record included an assessment of maternal depression/depressive symptoms prior to or concurrent with the self-regulation assessment.

### Study coding

Included studies were coded by the first author (a doctoral candidate in developmental and clinical psychology) or a trained research assistant (an undergraduate in child development) for the following variables, which were used in moderator analyses or to characterize the studies: 1) study information and sample characteristics, 2) maternal depression information, 3) infant self-regulation information, and 4) data for calculation/estimation of effect sizes. Twenty percent of records were second-coded to determine interrater reliability of each code used in moderator analyses. Additional information on specific coding procedures is provided in the Supplemental Methods.

#### Study information and sample characteristics

##### Potential Moderators.^7^

***Infant Sex*** (Cohen’s κ = .84). Percent of infants in the sample who were female, centered at 50%.

***Infant Age at Assessment of Self-Regulation*** (*κ* = .96). Infant age in months at the time of self-regulation assessment, mean-centered.

***Type of Sample*** (*κ* = .72). Community samples were intended to represent the broader general community, i.e., the researchers did not aim to recruit mothers with psychopathology. Clinical samples comprised participants recruited for clinical levels of maternal psychopathology or heightened psychological distress.

##### Additional descriptive codes.

***Mother Race and Ethnicity.*** Mother race and ethnicity, however it was reported in each study.

***Maternal Age.*** Mean and/or range of maternal age in years.

***Parity.*** Percent of mothers who were primiparous.

***Country of Study***. Country where data collection occurred.

#### Maternal depression information

##### Potential moderators.

***Maternal Depression Method*** (*κ* = 1.00). We coded the method as “self-report questionnaire,” “other-report questionnaire,” “diagnostic interview,” “extracted from medical chart,” “observational coding”, or “other.”

***Maternal Depression Timing*** (*κ* = .95). We coded timing as “lifetime history,” “pre-pregnancy,” “pregnancy,” “postnatal,” or “other.” Only pregnancy and postnatal had sufficient frequency to be included in moderator analyses.

***Type of Depression Score*** (*κ* = 1.00). We coded whether maternal depression scores were “binary” (e.g., presence or absence of a diagnosis of depression), “other categorical” (e.g., low, moderate, and high levels of depression), or “continuous.” The included effect sizes only used binary and continuous scores.

***Reliability of Depression Measure*** (*κ* = .88). Reliability metrics (e.g., Cronbach’s *α*, Cohen’s *κ*) were not directly comparable, so we created a *post-hoc* binary (adequate/inadequate) reliability variable using the following thresholds: Cronbach’s *α* ≥ .70 (Tavakol & Dennick, 2011), Cohen’s *κ* ≥ .60 (McHugh, 2012), and intraclass correlation coefficient ≥ .75 (Koo & Li, 2016). All included effect sizes that reported reliability used measures with adequate reliability; thus, we could not examine this moderator.

##### Additional descriptive codes.

***Measure of Maternal Depression***. The name of the maternal depression measure used.

#### Infant self-regulation information

##### Potential moderators.

***Self-Regulation Method*** (*κ* = 1.00). We coded the method as “questionnaire,” “observational coding,” “clinician assessment,” “interview,” “eye-tracking task,” or “other.”

***Reliability of Self-Regulation Measure*** (*κ* = 1.00). We created a *post-hoc* binary (adequate/inadequate) reliability variable using the following thresholds: Cronbach’s *α* and McDonald’s *ω* ≥ .70 (Tavakol & Dennick, 2011), Cohen’s *κ* ≥ .60 (McHugh, 2012), Intraclass Correlation Coefficient ≥ .75 (Koo & Li, 2016), and percent rater agreement ≥ 70.

***Independence of Self-Regulation Measure*** (*κ* = .96). We coded whether each self-regulation measure captured regulatory strategies employed independently by the infant or co-regulatory processes. See Supplemental Table S1 for details. Levels of this code, as well as the other codes indexing self-regulation conceptualization, are described with examples in Supplemental Table S1.

***Trait- or Process-Oriented Measure of Self-Regulation*** (*κ* = .94). We coded whether self-regulation was conceptualized as a trait-like aspect of infant temperament or as a regulatory process.

***Context of Self-Regulation Assessment*** (*κ* = 1.00). We coded whether regulation was assessed in the context of an acute stressor or not.

##### Additional descriptive codes.

***Self-Regulation-Related Construct Assessed.*** We coded which self-regulation-related construct was assessed for each effect size.

***Measure of Self-Regulation.*** We coded the infant self-regulation measure for each effect size.

##### Effect size data

We coded unadjusted bivariate Pearson’s product moment correlation coefficients or the information needed to estimate them from the following unadjusted statistics: point-biserial correlation coefficients, standardized *β*, frequencies from crosstab frequency tables, means and standard deviations, *t*-tests, *F*-tests, contingency tables, *χ*-squares, or exact significance levels. We estimated effect sizes using the formulas provided by Lipsey & Wilson (2001) via Wilson’s online effect size calculator (Wilson, 2023).^8^ We also recorded the analytic sample size for each effect size.

### Data analysis

#### Characteristics of included studies and effect sizes

We first examined characteristics of each included study and effect size, including study-level sample characteristics (e.g., sample type, demographics), characteristics of the measure of maternal perinatal depression, and characteristics of the measure of infant self-regulation.

##### Risk of Bias Assessment.

As part of our characterization of included studies, we considered various study characteristics related to risk of bias. First, we considered demographics of the participants represented in the studies (race and ethnicity, country of study, ages of included mothers and infants) to assess specificity or generalizability of findings to the broader population. We also considered various methodological characteristics, including whether the selected measures were previously established in the field and reliable in the present sample. Further, we considered the methods used for both maternal depression and infant self-regulation, which allowed us to consider potential issues such as shared reporter bias.

#### Effect size estimation

To synthesize effect sizes, we used zero-order Pearson product moment correlation coefficients, which estimate the rank-order association between two continuous variables (Lipsey & Wilson, 2001). We transformed Pearson product moment correlation coefficients using Fisher’s *Z*_*r*_-transformation to correct bias in the standard errors used to estimate inverse variance weights (Hedges & Olkin, 1985). We back-transformed the final pooled effect size for interpretation. We used the *metafor* package version 4.4–0 (Viechtbauer, 2010) in R (R Core Team, 2021) to apply the Fisher’s *Z*_*r*_-transformation to effect sizes and estimate inverse variance weights.

#### Synthesis of the overall association between maternal perinatal depression and infant self-regulation

For our primary analysis, we used a three-level random effects multilevel model. We selected a random-effects model because we hypothesized there would be systematic heterogeneity in the underlying true effect sizes represented by the effect sizes included in the meta-analysis due to methodological and conceptual differences (Harrer et al., 2021). We selected a three-level model to account for interdependence of effect sizes, as many of the records included multiple effect sizes, thus violating the assumption of statistical independence. Including a third level accounted for this interdependence (effect sizes nested within studies) and allowed us to retain all effect sizes (Harrer et al., 2021). We estimated *τ*^2^, the variance of the distribution of true effect sizes, using the restricted maximum-likelihood estimator, which performs well in meta-analyses synthesizing continuous effect sizes (Langan et al., 2019; Veroniki et al., 2016). To quantify precision of the synthesized effect size, we calculated the 95% confidence interval (CI). Finally, we examined whether a three-level model provided a better fit to the data than a two-level model using a likelihood ratio test (Harrer et al., 2021). We used the *metafor* package version 4.4–0 (Viechtbauer, 2010) in *R* for effect size synthesis.

#### Examine potential moderators of the association between maternal perinatal depression and infant self-regulation

After estimating an overall pooled effect size, we examined whether there was significant between-study and/or between-effect size heterogeneity in the estimate using Cochran’s *Q*, which tests whether the variation in the effect sizes in a meta-analysis exceeds the amount of variation expected if there were no between-effect size or between-study heterogeneity (e.g., if all variation were due to sampling error; Harrer et al., 2021). We also quantified the proportion of variance due to sampling error vs between-study and between-effect-size variation using a multilevel version of the *I*^2^ statistic (Cheung, 2014). By convention, *I*^2^ values of 25, 50%, and 75% represent low, moderate, and high heterogeneity, respectively (Migliavaca et al., 2022). Further, we calculated the 95% prediction interval (PI), which provides a range into which we can expect the effects of future studies in similar contexts to fall (IntHout et al., 2016). We used version 0.1.0 of the *dmetar* package (Harrer et al., 2021) in *R*.

For moderator analyses, we estimated separate three-level mixed-effects meta-analytic models with each moderator as a predictor using the *metafor* package version 4.4–0 (Viechtbauer, 2010) in R. For each moderator model, effect sizes with missing data on the relevant moderator were excluded. For categorical moderators, we did not include categories with fewer than *k* = 5 effect sizes in moderator analyses. Results of moderator analyses can be interpreted as follows: For both continuous and categorical moderators, the intercept is the expected effect size when the value of the moderator is zero (for categorical moderators, the value for the reference group). The unstandardized regression coefficient provides the estimated difference in the effect size for a one-unit difference in the value of the moderator (for categorical moderators, the difference in expected effect size between reference and comparison groups). We did not conduct a *priori* power analyses for overall or moderator effects.

#### Examination of publication bias and influential cases

To evaluate publication bias, we created a funnel plot and used Egger’s regression asymmetry test (Egger et al., 1997) in the *metafor* package version 4.4–0. Additionally, to explore our data for possible influential cases (Viechtbauer & Cheung, 2010), we calculated Cook’s distance for effect sizes and studies and standardized DFBETA values. As *a priori* guidelines, we considered Cook’s distances of > .45 or standardized DFBETA values > 2/√*k* to be evidence of potentially influential statistics (Harrer et al., 2021; Viechtbauer & Cheung, 2010). In the case of influential cases, we conducted sensitivity analyses without potential outliers to examine the robustness of our conclusions to potential influential cases.

## Results

### Characteristics of included studies and effect sizes

#### Overview of included studies

A total of 68 records, which comprised 193 effect sizes, were included in this meta-analysis (see Table A1 in the Appendix for included records). Analytic sample sizes of included effect sizes ranged from 11 to 5,728, with an average analytic *n* of 244 mother infant dyads.

#### Study information and sample characteristics.

See Supplemental Table S2 for descriptive statistics of variables coded to characterize included records and for moderator analyses. Sample demographics and data collection country varied across studies. Specifically, of the 50 (74%) records that provided information on the race and/or ethnicity of their samples, 34 (68%) comprised 50% or greater White participants. While most records (54%) described studies conducted in the United States, 15 other countries (primarily European) were represented in the meta-analysis. Mothers in the studies ranged in age from 15 to 51 years. Most studies included only adult mothers; of the studies that reported the age range of their participants, only three included mothers under 18 years of age. On average, studies included slightly more than half (65%) primiparous mothers. Seven studies limited participation to only primiparous mothers. There was limited variability in the proportion of male and female infants in the included samples, with all samples approximately evenly split between male and female infants (range: 40 – 63% female). Across included effect sizes, infant age ranged from assessment within 24 hours of birth to an average age of to 16.10 months. Finally, most effect sizes (72%) were from community samples.

#### Maternal depression information.

Next, we examined characteristics of the maternal depression assessments. Most (*k* = 109) effect sizes described associations between maternal postnatal depression and infant self-regulation. Most depression measures were self-report questionnaires (*k* = 156), and most used continuous measures of depression (*k* = 150). Among the 106 effect sizes for which reliability (internal consistency or interrater reliability) of the measure of maternal depression was reported, all reported adequate reliability. Finally, regarding measures used to assess maternal depression, the Edinburgh Postnatal Depression Scale (EPDS; Cox et al., 1987) was by far the most common (*k* = 87), followed by the Center for Epidemiologic Studies-Depression Scale (CES-D; Radloff, 1977; *k* = 26).

#### Infant self-regulation information.

Next, we examined characteristics of infant self-regulation assessments. Emotion regulation and self-regulation were the most frequently assessed constructs (*k* = 37 each), followed by regulation (*k* = 34). For most effect sizes, infant self-regulation was assessed with parent-report questionnaires (*k* = 104), but assessment using observational coding of structured tasks was also common (*k* = 66). The most common measure used to assess infant self-regulation was the Infant Behavior Questionnaire-Revised (IBQ-R; Gartstein & Rothbart, 2003; *k* = 87), although there was wide variability in the factors or subscales of the IBQ-R selected (e.g., Orienting/Regulatory Control factor, Negative Affectivity factor, Decreased Vocal Reactivity subscale). The most common observational coding paradigms were mother-infant structured- or free-play interactions (*k* = 25) and tasks from the Laboratory Temperament Assessment Battery (LabTAB; Goldsmith & Rothbart, 1992; *k* = 18). Self-regulation measure reliability was provided for 93 effect sizes, and of these, most (*k* = 71) showed adequate reliability. Regarding self-regulation conceptualizations, most effect sizes used measures that focused on combined approaches (*k* = 80) or independent infant contributions (*k* = 79) to self-regulation and did not assess self-regulation during stress tasks (*k* = 156). More than half of effect sizes used a measure that captured trait-like self-regulation (*k* = 107).

### Synthesis of the overall association between maternal perinatal depression and infant self-regulation

**Figure 2.**
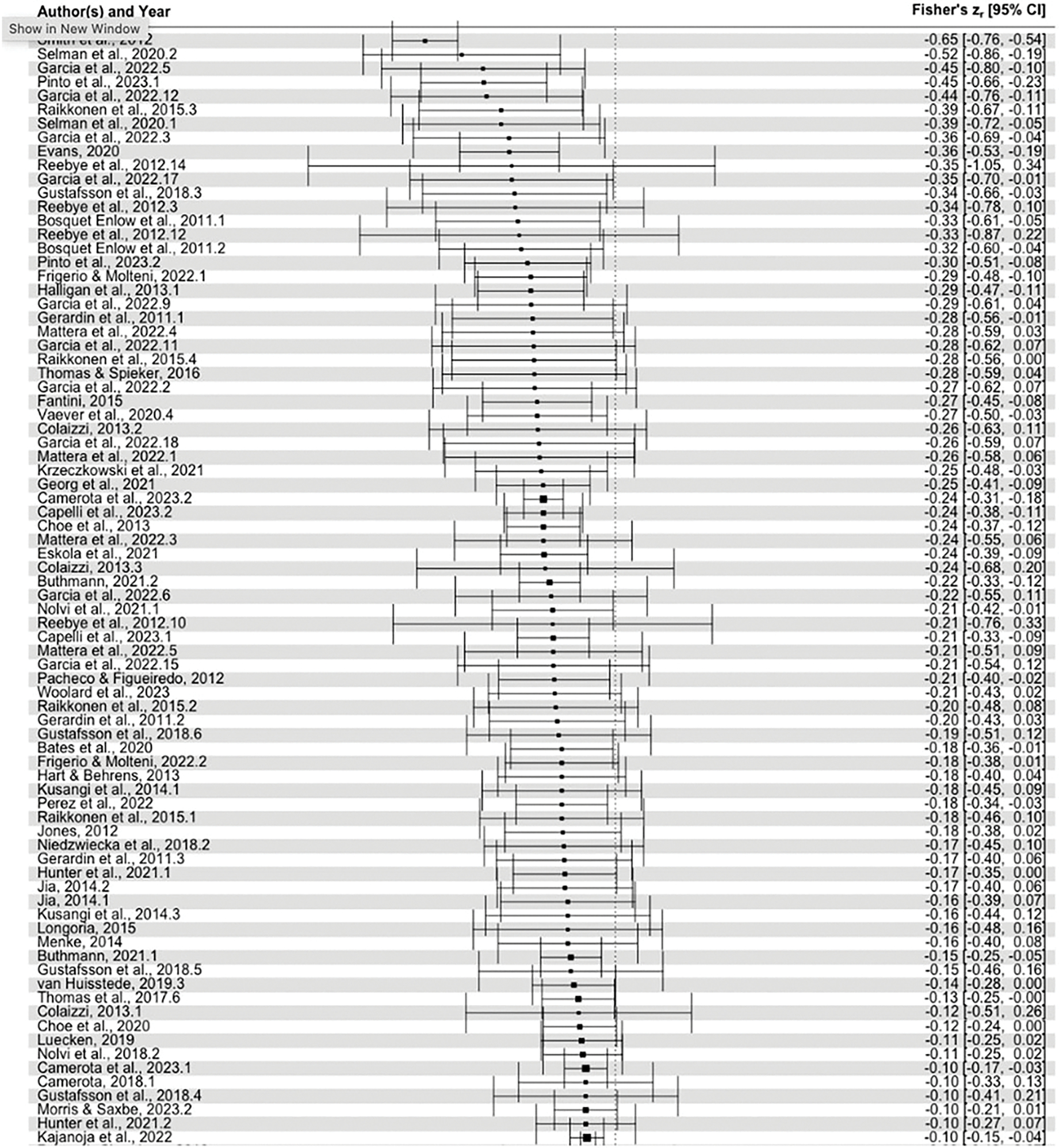

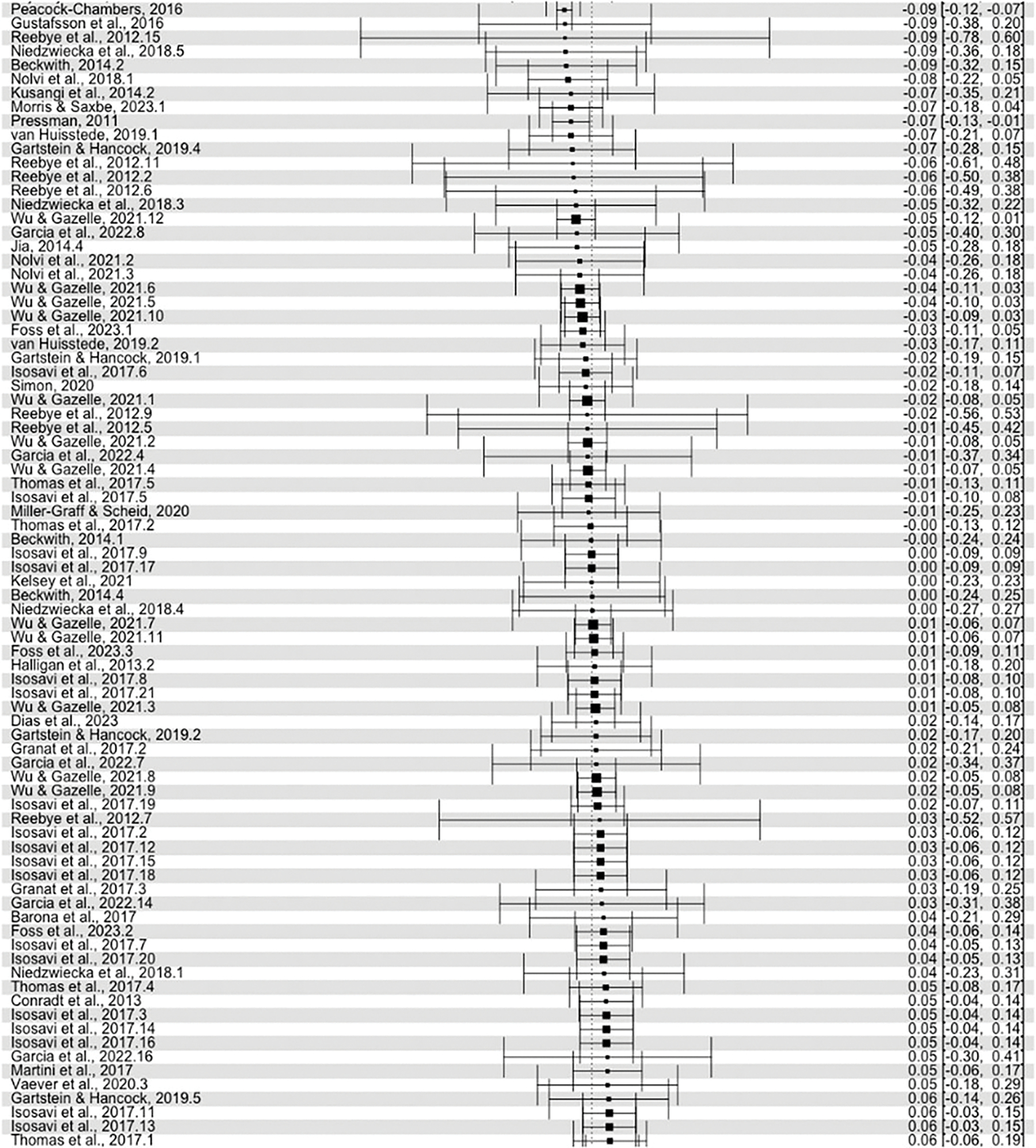

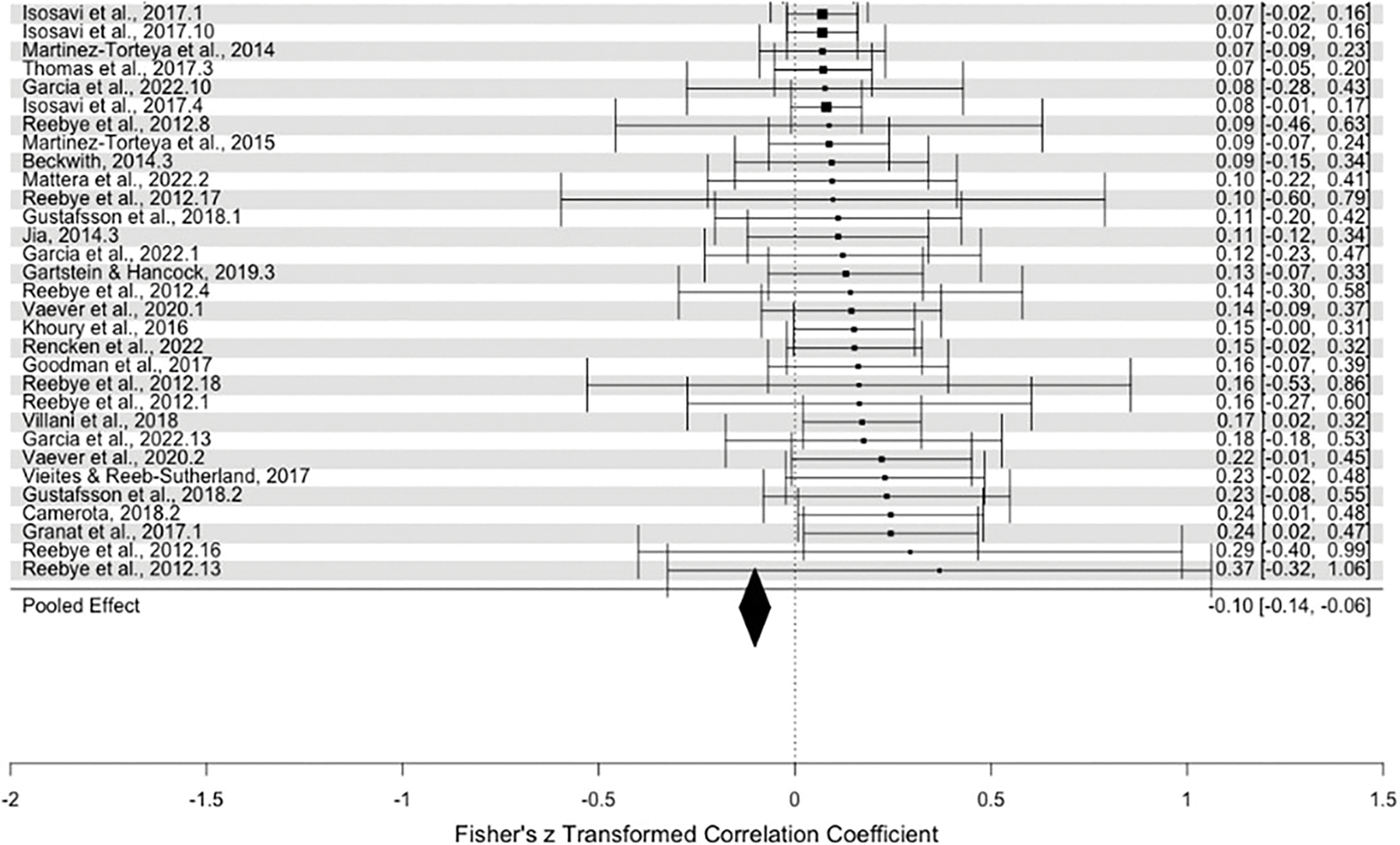
Forest plot of included effect sizes and overall pooled effect size of the association between maternal perinatal depression and infant self-regulation. *Note*. This figure shows estimated effect sizes (Fishers *Zr*-transformed correlation coefficients) and 95% confidence intervals of the included studies, as well as the overall pooled effect size. Stronger negative associations indicate that higher levels of maternal depression were associated with lower infant self-regulation.

As hypothesized, overall, higher levels of maternal perinatal depression were significantly associated with lower infant self-regulation (*r* = −.10, 95% CI = −.14, −.06, *p* < .001; Figure 2). The three-level model fit the data better than a two-level model with level-3 heterogeneity constrained to zero (*χ*^2^(1) = 90.61, *p* < .001).

### Examine potential moderators of the association between maternal perinatal depression and infant self-regulation

We next examined heterogeneity in the overall effect size using multiple indices. The *Q*-statistic test for heterogeneity suggested true effect size differences in the data (*Q* (192) = 577.44, *p* < .001). Further, the PI ranged from *r* = −.36 to *r* = .17, indicating that levels of heterogeneity suggest that positive associations (i.e., in which more maternal depression is associated with more infant self-regulation) not due to sampling error cannot be ruled out for future studies (Harrer et al., 2021). The multilevel *I*^2^ suggested about 18% of variance was due to sampling error, less than 1% of variance was due to within-study characteristics, and about 82% of variance was due to between-study characteristics. Thus, there was substantial heterogeneity not due to sampling error, and most of this heterogeneity was at the between-study level, indicating follow-up moderator analyses to examine this heterogeneity were warranted.

Results of moderator analyses are presented in Table 2.

#### Study information and sample characteristics

None of the variables describing study or sample characteristics (i.e., infant sex, infant age, or sample type) significantly moderated the association (see Table 2, all *ps* > .305).

#### Maternal depression information

Similarly, none of the maternal depression-related variables (i.e., method, timing, or score type) significantly moderated the association (see Table 2, all *ps* > .147).

#### Infant self-regulation information

Several infant self-regulation-related variables moderated the association, although none remained significant after multiple comparisons correction. First, the magnitude of the association between maternal depression and infant self-regulation depended on the method used to assess infant self-regulation. The estimated effect size was smaller and not significantly different from zero when infant self-regulation was assessed with observational coding (*r*_*IntObservational*_ = −.04, 95% CI = −.09, .02, *p* = .185) compared to questionnaires (*r*_*IntQuestionnaire*_ = −.12, 95% CI = −.16, −.08, *p* =< .001) and clinician assessments (*r*_*IntClinician*_ = −.15, 95% CI = −.23, −.07, *p* = < .001). The association did not differ between questionnaires and clinician assessments. The other two significant moderators were among the exploratory codes aimed to capture self-regulation conceptualization. Effect sizes based on trait-focused self-regulation measures (*r*_*IntTrait*_ = −.13, 95% CI = −.17, −.08, *p* = < .001) showed stronger associations than effect sizes based on process-focused self-regulation measures (*r*_*IntProcess*_ = −.07, 95% CI = −.12, −.02, *p* = .005). Further, effect sizes based on self-regulation measures in stressor contexts (*r*_*IntStressor*_ = −.04, 95% CI = −.10, .02, *p* = .221) showed weaker, non-significant associations than those based on measures in non-stressor contexts (*r*_*IntNotStressor*_ = −.11, 95% CI = −.15, −.07, *p* = < .001).

In sum, overall, more maternal depression was associated with lower infant self-regulation, and this pooled effect showed substantial heterogeneity. Effect sizes differed across levels of three moderators: self-regulation method, whether the self-regulation method was trait- vs. process-focused, and whether self-regulation was assessed in a stressor context.

#### Examination of publication bias and influential cases

**Figure 3.**
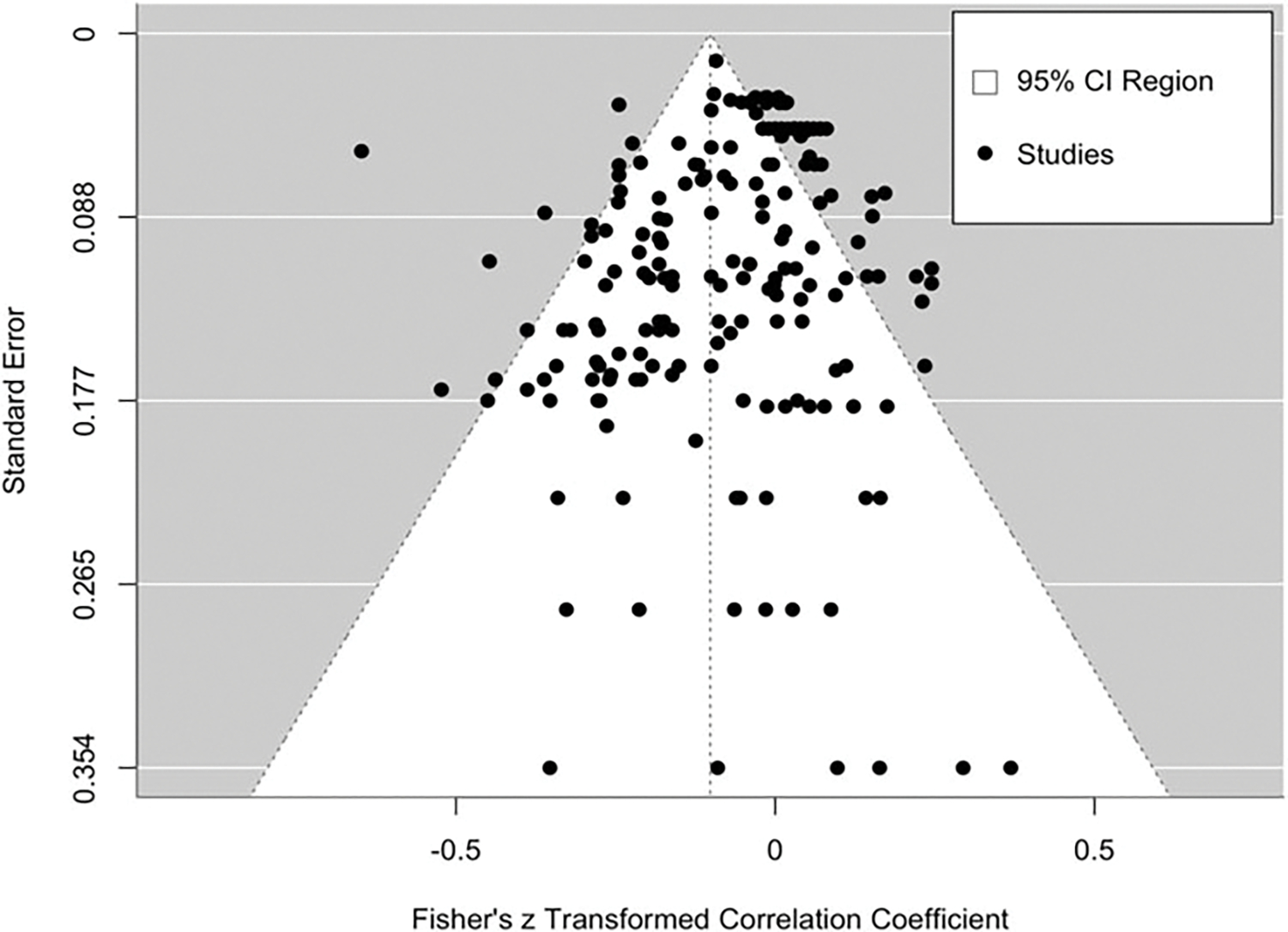
Funnel plot providing visualization of potential small study bias.

Figure 3 shows the funnel plot for the meta-analysis. Visual inspection did not suggest substantial asymmetry. Consistent with this visual inspection, the intercept of Egger’s regression test was not significantly different from zero (*B*_*0*_ = 0.02, *SE* = .02, *p* = .197). These findings do not raise concerns for publication bias.

We used multiple methods to explore our data for cases that may have undue influence on our results. Based on Cook’s distance, we did not identify any such cases. However, standardized DFBETA values suggested eight effect sizes (Conradt et al., 2013; Evans, 2020; Goodman et al., 2017; Khoury et al., 2016; Rencken et al., 2022; Smith et al., 2012; Vieites & Reeb-Sutherland, 2017; and Villani et al., 2018) may function as influential cases based on our *a priori* guidelines. We ran eight separate sensitivity analyses each leaving out one of these effect sizes. All sensitivity models indicated a significant negative association between maternal depression and infant self-regulation, consistent with the primary analysis, and suggesting limited impact of these cases on substantive conclusions.

## Discussion

### Meta-analytic findings

The overall meta-analytic effect suggested that higher levels of maternal perinatal depression were associated with modestly lower infant self-regulation. This finding is consistent with the leading theoretical model of the intergenerational transmission of depression (Goodman & Gotlib, 1999) positing that maternal depression undermines offspring self-regulation, and that these resulting self-regulation difficulties are a vulnerability increasing risk for offspring depression. Indeed, difficulties with self-regulation are implicated as a key process in depression onset and maintenance (e.g., Joormann & Stanton, 2016), further underscoring the importance of identifying and intervening upon early exposures that may undermine self-regulation.

Importantly, multiple approaches for quantifying heterogeneity indicated significant heterogeneity in the overall effect. This heterogeneity motivates examining moderators of the overall association. For example, the PI suggests that some levels of some moderators may yield group estimates for which higher levels of maternal depression are associated with more infant self-regulation. None of our selected moderators produced such results, but others that we did not consider may, which would have important theoretical implications for understanding nuances of this association.

Several methodological variables did moderate the overall effect, although none survived multiple comparisons correction. First, the infant self-regulation assessment method moderated the strength of the association, with effect sizes based on observational coding showing smaller (null) associations than effect sizes using questionnaires and clincian assessments. Questionnaire measures of self-regulation were typically completed by mothers, and it is possible the associations based on these measures were inflated by a negative reporting bias among mothers with depression or by a more general shared informant bias. Alongside this potential bias, it is also important to consider a strength of questionnaire measures filled out by mothers; namely, when responding to questionnaire measures, mothers have a considerable amount of data, given the amount of time they spend with their infant, on which to base their ratings. Clinician assessments (which rely on the direct observations of trained clinicians who are often masked^9^ to maternal depression levels) and observational coding are less likely to be affected by shared informant biases. However, while observational approaches are often considered the gold standard for observable psychological constructs, these approaches also have limitations. For example, scores on one-time observational measures capture a very small sample of infant behavior in a highly specific context and are likely also influenced by factors other than self-regulatory capacities (e.g., infant state, time since sleep or feeding).

Thus, observational data collected by trained research staff typically include highly standardized protocols and rigorous standards of inter-rater agreement. These are clear strengths. However, they may sacrifice some ecological validity, given the typically more contrived observational context, the limited observations of infants over relatively short timeframes, and the idiosyncratic noise that can arise with such snapshots in time – particularly with infants. In contrast, parental reports of infant behavior have the benefits of extended observations, occurring across more varied contexts, yet may be less objective and potentially reflect reporter biases. Indeed, this is particularly concerning for studies of maternal depression, where mothers’ own symptoms may color their observations of their infants’ behavior. The fact that we observed very similar findings between maternal and clinical observations – the latter often masked to maternal depression – may help to somewhat mitigate these concerns. For example, in addition to being statistically indistinguishable (which could potentially reflect the imprecision of the estimates), the effect sizes were quite similar in absolute terms. Nevertheless, all findings should be weighed with respect to their methodological strengths and limitations.

The additional significant moderators were whether the effect size relied on a trait-like measure of self-regulation and whether the self-regulation measure was assessed in a stressor context. It is important to note that these two codes overlap in important ways. While it would be possible for a trait-like measure to be coded as occurring in a stressor context, this did not occur in our dataset. However, the effect sizes coded as process-oriented were split between stressor and non-stressor contexts. These findings suggest that maternal depression may be more strongly related to general, trait-like self-regulation than to in-the-moment regulatory strategies. As with the self-regulation assessment method moderator, it is possible that a shared-reporter bias could in-part explain this finding, as most trait-like self-regulation measures were mother-reported. However, it is also possible that there are real substantive differences in the way maternal depression shapes trait-like versus in-the-moment regulation. Indeed, previous research indicates that associations between self-regulation assessed in a trait-like manner and self-regulation assessed in the moment are modest (Aureli et al., 2015), suggesting that these two assessment approaches capture somewhat related but largely distinct phenomena, which further underscores the importance of separating these two constructs to better understand their development and sequelae.

It is noteworthy that many variables that we expected would moderate the association did not. There are several potential explanations for these null findings, some methodological and some substantive. One important methodological consideration is that, given the relatively modest number of studies we were able to include in the meta-analysis, we were likely underpowered to detect some moderating effects. Hempel and colleagues (Hempel et al., 2013) note that few meta-analyses, particularly those in which values of the moderator are not equally distributed across categories, are powered to detect moderator effects. The distributions of several of our null moderators may have limited our ability to detect effects (i.e., the range of infant sex was quite restricted and the distributions of sample type, maternal depression method, maternal depression score type, and reliability of self-regulation measure were very uneven). One moderator that demonstrated a more even distribution and yielded a null result was maternal depression timing. However, given considerable stability in maternal perinatal depression (e.g., DiPietro et al., 2008) and our use of unadjusted bivariate correlation coefficients, this meta-analytic study was likely not well-suited to isolate timing effects.

Notably, the overall pooled effect size and effect sizes within moderator subgroups were small (Cohen, 1988). However, when considering the many influences that shape development in the first years of life, it is unsurprising that one specific exposure would have only a small effect (Götz et al., 2021). Importantly, many established, clinically important effects are small in magnitude (e.g., Meyer et al., 2001; Owens et al., 2021). Thus, small effect sizes such as this one, when estimated with precision, are expected and may still be practically meaningful.

### Quality of existing literature

#### Sociodemographic considerations

While there was variability in sociodemographic characteristics of the included studies, there were also notable gaps. First, 68% of studies comprised majority White participants, and over half were conducted in the United States, with most others conducted in Europe. There were notable exceptions, including Luecken and colleagues’ (2019) study with Mexican and Mexican American dyads and Isosävi and colleagues’ (2017) study with Palestinian dyads. With the goal of producing a broadly generalizable literature and given that both experiences of maternal depression (e.g., Bashiri & Spielvogel, 1999; Maxwell et al., 2019) and self-regulation development (e.g., LeCuyer & Zhang, 2015) may differ across racial and ethnic groups and cultural contexts, examining this association in more diverse or non-White samples and in non-Western contexts is critical for informing conceptual models and more broadly applicable clinical considerations.

Another limitation of the literature includes a focus on adult mothers. While including adolescent mothers requires additional ethical considerations (e.g., parental consent), given the stressors of adolescent parenthood, this population may be particularly vulnerable to perinatal psychopathology (Jeon et al., 2024), and understanding risk and protective processes for adolescent mothers and their infants has critical prevention/intervention implications. Further, we were unable to code the percentage of mothers in each sample who were their infants’ biological mothers because of limited reporting of this characteristic. While a few studies restricted their sample to biological mothers only, no other included studies reported this information. We assume most studies included primarily biological mothers, consistent with the broader perinatal mental health literature, which includes limited consideration of adoptive and foster parents’ mental health (McKay et al., 2010). Clear reporting on this characteristic would aid understanding of mother-infant mental health, with implications for supporting adoptive and foster mothers – many of whom may be parenting infants with complex needs related to prenatal and early life trauma and stress – and for understanding mechanisms of intergenerational risk transmission.

#### Maternal depression assessment

The included literature used well-established and validated self-report questionnaires or diagnostic interviews to assess maternal perinatal depression. While diagnostic interviews are considered the gold standard for assessing clinical levels of psychopathology, considering depressive symptoms using psychometrically sound self-report questionnaires also provides important information about subthreshold symptoms. Previous research suggests both clinical psychopathology assessed diagnostically and subthreshold symptoms assessed with continuous rating scales have implications for parenting and offspring functioning (e.g., Goodman et al., 2020; West & Newman, 2003). Thus, it is important to continue examining subthreshold and clinical perinatal depression. Notably, we were only able to code reliability of the maternal depression measure for about half of the included effect sizes, limiting our ability to consider reliability in the included samples. However, consistent with the use of validated assessments, for those effect sizes with reliability information, all exceeded our *a priori* thresholds indicating adequate reliability.

#### Self-regulation assessment

Consistent with the broader literature on infant self-regulation and our search strategy, our search demonstrated use of a wide range of methodologically and conceptually variable infant self-regulation assessment approaches. There is disagreement about the extent to which different outcome measures can be meta-analytically combined (e.g., Lipsey & Wilson, 2001). We opted for an inclusive approach, given that self-regulation-related terminology is often used inconsistently and interchangeably across studies (e.g., within the included studies, the orienting/regulatory capacity factor of the IBQ-R was variously described as assessing regulation, self-regulation, emotion regulation, and effortful control). As such, in the present review, infant self-regulation was assessed with measures of regulation, stress regulation, emotion/mood regulation, mutual/co-regulation, self-regulation, attentional control, state regulation, behavioral regulation, fear regulation, and inhibition. Various methods were also used, including observational coding of infants’ attention, behavior, and emotion during stress tasks or social interactions; parent-report questionnaires; structured assessments conducted by trained clinicians; and parent interviews. Notably, self-regulation measure reliability was only reported for about half of included effect sizes (of those that did report reliability information, 77% met our *a priori* thresholds for adequate reliability). There was little consistency in which measures showed inadequate reliability. For example, in some studies the orienting/regulatory control factor of the IBQ-R showed adequate reliability, while in other studies it did not, emphasizing the importance of examining reliability *within a given sample*, rather than relying solely on previous evidence of reliability. Given the variability in approaches to measuring self-regulation and the frequent use of novel or recently developed measures, it is critical that authors provide information about the reliability of their measures, both for results interpretation and to guide future measure selection.

### Limitations of the meta-analysis

While this meta-analysis addresses an important gap in the literature, several limitations must be considered. Given the high prevalence of maternal perinatal depression (Bauman et al., 2020), the importance of self-regulation for functioning across several domains (e.g., Aldao et al., 2016; Best et al., 2011; Fitzsimons & Finkel, 2011), and the strong theoretical rationale for an association between the two, it is unsurprising that our search yielded a relatively large body of recent literature examining this association (129 records). However, we were unable to include 61 records because adequate information for effect size estimation was not reported. This underscores the importance of reporting descriptive statistics and zero-order correlations of all study variables to facilitate cumulative science.

An additional limitation of the current study, particularly in light of null moderator results, is that we did not conduct *a priori* power analyses to determine the scenarios under which moderator analyses would be powered to detect true effects, as this is not often done in meta-analytic reviews. However, Hedges & Pigott (2004) provide guidelines to conduct such power analyses that can be applied *a priori* to future work to help differentiate true null moderator effects from false negatives.

#### Implications and future directions

Despite these limitations, this meta-analysis has two important implications. First, the association between maternal perinatal depression and infant self-regulation emphasizes the interconnectedness of mother-infant dyads during this sensitive developmental period. Whether the identified association represents a causal pathway from maternal depression to infant functioning or arises from other familial and/or contextual risk factors, our finding indicates that efforts to support both maternal and infant outcomes are warranted among dyads experiencing maternal depression. Second, this finding suggests important future research directions. While clear and specific policy and clinical implications require causally informed data, establishing support for this association grounds future research examining causal processes and is thus a critical step for identifying policy and clinical implications.

Potential causal pathways linking maternal perinatal depression and infant self-regulation are described in Goodman and Gotlib’s (1999) seminal model of the intergenerational transmission of depression. The model conceptualizes self-regulation impairments as one vulnerability observable in infants and children of mothers with depression that increases later risk of depression. Indeed, children of parents experiencing depression are at three-fold risk of developing depression themselves (Weissman et al., 2006), underscoring the importance of understanding early risk indicators in this population.

Two pathways in Goodman & Gotlib’s model suggest a causal effect of maternal depression on infant self-regulation. First, physiological differences in the maternal and fetal environments in the context of maternal depression may impact developing fetal and infant stress systems. Stress regulatory systems that underlie early physiological regulation, such as the ANS and the HPA axis, begin to develop in-utero (Cerritelli et al., 2021; Howland et al., 2017; Irwin et al., 2021). These systems develop rapidly and are sensitive to environmental impacts, such as levels of maternal and placental stress hormones or maternal inflammation (Aboustate & Baune, 2020; Henrichs & Van den Bergh, 2015; Irwin et al., 2021), which are amplified in the context of maternal depression. Indeed, fetuses and infants of mothers who experience prenatal depressive symptoms tend to show differences in physiological regulation (Field et al., 2004; Galbally et al., 2020; Kinsella & Monk, 2009; Monk et al., 2011). Thus, the observed association of elevated maternal depression and lowered infant self-regulation may arise in part via maternal prenatal physiological differences that affect fetal and infant physiological regulation.

Second, after birth, the development of self-regulation continues in the context of caregiver-infant interactions (Gianino & Tronick, 1988). The co-regulatory social interactions that support the infant’s developing self-regulation rely on the infant’s physiological regulatory capacities *and* the caregiver’s ability to interpret and respond appropriately to their infant’s communicative signals (Beeghly et al., 2016), often referred to as sensitive parenting/caregiving (Ainsworth, 1969). More sensitive caregiving is associated with more self-regulation in infancy, toddlerhood, and early childhood (Augustine et al., 2018; Bernier et al., 2010; Blair et al., 2006; Camerota et al., 2015; Fay-Stammbach et al., 2014; Hofstee et al., 2022; Samdan, 2020). Notably, a large body of evidence also indicates that maternal depression is associated with more difficulty engaging in sensitive caregiving behaviors (Bernard et al., 2018; Goodman et al., 2020; Mah, 2016). Infants of mothers experiencing depression during the postnatal period may thus show difficulties with self-regulation in part due to reduced sensitive caregiving.

Importantly, while these two mechanisms are most directly implicated in explaining a potential causal effect of maternal perinatal depression on infant self-regulation, other factors may explain the observed association between maternal perinatal depression and infant self-regulation (Goodman & Gotlib, 1999). For instance, shared genetic factors underlying a liability toward dysregulation (Bridgett et al., 2015) or contextual stressors driving both maternal depression and infant dysregulation may also explain the association observed in this meta-analysis.

To better understand the association between maternal depression and infant self-regulation, it is necessary to consider all of these pathways and their likely co-occurrence. Given confounding of prenatal environmental effects, parenting behaviors, genetic effects, and stressful life circumstances in observational family studies, teasing apart the relative causal contributions of each of these pathways proves difficult. However, examining relevant questions with a combination of thoughtful and rigorous methodological approaches including twin, family, and adoptive designs; other natural experiments; intervention studies; and statistical approaches that control for potential confounders provides a way forward (e.g., Davis et al., 2018; Rutter, 2007; Wilson & Rhee, 2022).

## Conclusions

In sum, the present meta-analysis synthesized the association between maternal perinatal depression and infant self-regulation across 193 effect sizes. We found a small, negative association indicating higher levels of maternal perinatal depression are associated with lower infant self-regulation. While notable limitations in this literature highlight important future directions, this association underscores the importance of supporting mother-infant dyads as a strategy for bolstering mental health and well-being for future generations.

## Supplementary Material

Supplementary Material

**Supplementary material.** The supplementary material for this article can be found at https://doi.org/10.1017/S0954579425100837.

## Figures and Tables

**Table 1.** Self-regulation-related constructs in the meta-analysls

Self-regulation	Cognitive Inhibition	Inhibition
Affect(ive) (dys)regulation	Cognitive self-regulation	Inhibitory control
Affect(ive) self-regulation	Cognitive (dys) regulation	Mood (dys) regulation
Attention(al) control	Co-regulation^a^	Mutual (dys) regulation^a^
Attention(al) (dys) regulation	(Dys)regulation^b^	Reactive control
Attention(al) self-regulation	Effortful control	Self-control
Attention(al) shifting	Emotion(al) (dys) regulation^c^	Set-shifting
Behavior(al) (dys) regulation	Emotion(al) self-regulation	State (dys) regulation
Behavior(al) (dis) inhibition	Emotion-related self-regulation	Stress (dys) regulation
Behavior(al) self-regulation	Executive attention	Updating
Biobehavioral regulation	Executive control	Working memory
Cognitive control	Executive function	
Cognitive flexibility	Fearful inhibition	

a We excluded effect sizes for which the measure of self-regulation assessed only *maternal* contributions to infant regulation.

b We did not include assessments of (dys)regulation that clearly did not relate to self-regulation (e.g., regulation of laws, regulation of genes).

c Or (dys)regulation of a specific emotion (e.g., fear regulation).

**Table 2.** Results of moderator analyses

		Intercept	Slope			
Moderator (Reference Group)	*k*	*B*	*B*	*95% CI*	*p*	Adj. *p*^a^
**Study Information and Sample Characteristics**						
Infant Sex^b^	151	−.10	.00	−.01, .01	.524	.673
Infant Age^c^	188	−.11	.00	.00, .01	.305	.543
Sample Type (Community)	193	−.10				
Clinical			−.01	−.08, .05	.669	.753
**Maternal Depression Information**						
Method (Self-Report)	186	−.11				
Diagnostic Interview			.06	−.05, .17	.299	.543
Timing (Pregnancy)	181	−.08				
Postnatal			−.04	−.08, .01	.147	.529
Score Type (Binary)	193	−.13				
Continuous			.04	−.03, .11	.296	.543
**Infant Self-Regulation Information**						
Method (Questionnaire)	188	−.12				
Observational Coding			.08	.03, .14	.003*	.054
Clinician Assessment			−.03	−.13, .06	.511	.674
Method (Observational Coding)	188	−.04				
Clinician Assessment			−.11	−.21, −.01	.028*	.126
Reliability (Inadequate)	93	−.12				
Adequate			.02	−.07, .11	.641	.753
Independence (Independent)	193	−.09				
Combined			−.02	−.06, .02	.324	.543
Distress			.00	−.05, .04	.888	.888
Bids to Mother			−.03	−.07, .01	.205	.543
Independence (Distress)	193	−.10				
Combined			−.02	−.05, .02	.332	.543
Bids to Mother			−.02	−.08, .04	.441	.662
Independence (Combined)	193	−.11				
Bids to Mother			−.01	−.06, .05	.820	.868
Trait/Process (Process)	193	−.07				
Trait			−.06	−.11, −.01	.027*	.126
Assessment Context (Stressor)	193	−.04				
Non-stressor			−.07	−.13, −.01	.020*	.126

*Note.* All moderator analyses were conducted in a meta-regression framework, with categorical moderators entered using dummy coding. The unstandardized coefficient of the intercept is the effect size when the moderator is zero (for categorical moderators, the effect size for the reference group). The unstandardized coefficient of the slope is the estimated change in the effect size for a one-unit change in the value of the moderator (for categorical moderators, the estimated difference in effect sizes between the reference and comparison groups). For categorical moderators, the reference groups are indicated in parentheses. For categorical moderators with greater than two levels, multiple models with different reference groups were estimated to present all possible comparisons.

a *p*-values adjusted for multiple comparisons using the Benjamini-Hochberg adjustment.

b Percent of the sample that is female, centered at 50%.

c Mean age of the sample in months, mean-centered.

* *p* < .05.

## Data Availability

We adhered to TOP Level 2 Guidelines. The data and code necessary to reproduce the analyses presented here, along with the coding documentation, are publicly accessible at the following URL: https://osf.io/jw7t2/overview?view_only=76cb058fad0e42c9b584ec4c675ba0a1.

## Footnotes

1 While we conceptualize self-regulation as referring to internally initiated processes, we note that “internally initiated” is not synonymous with “independent.” Particularly in infancy, self-regulation encompasses internally initiated bids for the regulatory support of important others (e.g., Khoury et al., 2016).

2 As evidence of this inconsistency, we had initially planned to use “self-regulation construct” (e.g., effortful control vs executive function vs behavioral regulation, etc.) as a moderator in our analyses. However, the results of our search revealed little consistency within levels of this planned moderator and substantial overlap between levels, suggesting that any results would be uninterpretable.

3 It is important to note that the contributions of other caregivers, including fathers, shape the development of self-regulation in important ways and can be similarly affected by depression (e.g., Cameron et al., 2016; Cheung & Theule, 2019). However, the existing literature and that screened for the present review focuses almost exclusively on *maternal* depression and infant self-regulation. Thus, we focused on maternal depression in the present review, but examining similar questions among other caregivers is an important avenue for future research. We also acknowledge that not all gestational parents identify as mothers. Our use of the words “mother” and “maternal” reflect that these terms were used to describe the participants in the studies included in our review.

4 While concurrent assessment does not clarify the directionality of an association, we retained studies that assessed our variables concurrently because it is possible in these cases that maternal depression influences infant self-regulation. We excluded only studies with temporal ordering that is inconsistent with our theoretical model (i.e., infant self-regulation assessed prior to maternal depression). To examine robustness of our results to the decision to include concurrent effect sizes, we ran a sensitivity analysis excluding these effect sizes. While the overall pooled effect decreased somewhat in magnitude, maternal depression remained significantly negatively associated with infant self-regulation (*r* = −.07, 95% CI = −.11, −.03, *p* = .001).

5 We initially planned and pre-registered to include articles that assessed self-regulation with a physiological indicator (e.g., salivary cortisol) given our conceptualization of physiological regulation as a component of self-regulation. However, review of the literature on physiological indicators of self-regulation revealed limited consensus on which patterns of physiological responding indicate more or less regulation, particularly given differences in assessment paradigms and data processing. For example, there is evidence that dysregulation of the HPA-axis can manifest as both hypo- and hyperreactivity (e.g., Gunnar & Quevedo, 2007). Thus, we were unable to include effect sizes using physiological measures in the meta-analysis (*n* = 12).

6 When relevant effect size information was not presented in the article or online supplement, we contacted corresponding authors to request effect size information. Consistent with the American Psychological Association guideline to retain records for 7 years, we contacted authors of articles published in the last 7 years. Thirty-four authors were contacted, 19 of whom responded and 11 of whom provided relevant statistics. Of those who responded but did not provide statistics, all indicated that they no longer had access to the data or did not have time to access the requested statistics.

7 We also intended to examine the percentage of mothers who were biological mothers as a moderator. However, this information was rarely reported.

8 For three effect sizes, only Spearman’s rho correlation coefficients were provided. Spearman’s rho cannot be converted to a Pearson’s correlation coefficient. However, to retain as many effect sizes as possible, we chose to treat these Spearman’s correlation coefficients as an approximation of the Pearson’s correlation coefficient and include them in analyses without any conversion. We also ran a sensitivity analysis without these three effect sizes. Substantive conclusions were identical with or without these effect sizes.

9 Of the 11 studies included in the meta-analysis that used clinician assessments, six reported that clinicians were masked to maternal depression information when assessing infant self-regulation (the other five studies did not specify whether clinicians were masked),
