# Supplementary material for: Maternal perinatal depression and infant self-regulation: A meta-analytic review
Source: Dev Psychopathol. Author manuscript; Available in PMC 2026 Jan 5. (PMC12766785; doi:10.1017/S0954579425100837)
Supplement: Supplementary Material [file NIHMS2133253-supplement-Supplementary_Material.docx]

**Supplemental Methods**

**Openness and Transparency**

We adhered to the MARS guidelines for meta-analytic reporting (Appelbaum et al., 2018) and the PRISMA 2020 guidelines for systematic reviews (Page et al., 2021). The pre-registration, dataset containing coded articles, coding instructions, and analytic code can be found on the Open Science Framework: https://osf.io/jw7t2/overview?view_only=76cb058fad0e42c9b584ec4c675ba0a1. Data were analyzed using *R*, version 4.4.1 (R Core Team, 2021) and the *metafor* package, version 4.4-0 (Viechtbauer, 2010). The present meta-analysis was pre-registered in Prospero on April 17, 2023.

***Deviations from Preregistration***

Over the course of the literature search, screening, coding, and analysis, there were deviations from preregistration. First, we conducted the database search using our preregistered search terms, but we searched directly from the listed databases rather than drawing our records from a database created during our larger self-regulation scoping review (as described in the preregistration). This change allowed us to update the search beyond the dates of the previous scoping review. We also preregistered to limit the search to peer-reviewed journal articles but removed this limit to include unpublished literature (e.g., dissertations). We did not include physiological assessments of infant self-regulation. We also altered our approach toward examining conceptualizations of self-regulation in moderator analyses. We initially intended to categorize conceptualization of self-regulation based on the terminology selected by the study authors. However, given the substantial heterogeneity in terminology applied across studies, we did not feel that this approach captured meaningful variation in conceptualizations of self-regulation. Thus, we created several *post-hoc* codes for use in exploratory moderator analyses. Finally, we pre-registered to use the Risk of Bias in Non-Randomized Studies-of Exposures (ROBINS-E) tool to assess risk of bias in the included studies (Higgins et al., 2023). However, we determined that it was not well-aligned with the types of studies included in the meta-analysis and instead used an informal approach to assessment of study quality. Finally, we assessed influential cases and small-study bias, which were not discussed in the pre-registration.

**Study Coding**

***Infant Sex***

We coded the percent of infants in the sample who were female. If this percentage was not reported directly, we estimated it from the percent male where possible. We centered percent female at 50% and entered it as a continuous moderator in analyses.

***Infant Age at Assessment of Self-Regulation***

We coded the age of the infants in the study at the timepoint when the self-regulation construct was assessed. When available, we coded mean age. If only an age range was provided, we coded the age range and used the average of the upper and lower bounds in moderator analyses. When neither mean nor range were provided, we coded a variable that we referred to as “target age.” The target age was the selected age at which the researchers aimed to assess infants. For example, we would code a target age of 2 months if the authors reported that infants were assessed at 2 months but did not report any more specific age statistics. For moderator analyses, we converted all infant ages to months. We used months corrected age (i.e., adjusted for prematurity) when relevant and provided. We mean-centered infant age and entered it in months as a continuous moderator in analyses.

***Type of Sample***

We characterized the sample type for each study as either “community” or “clinical.” Community samples were defined as samples intended to represent the broader general community, i.e., the researchers did not aim to specifically recruit mothers with clinical levels of psychopathology (although they likely included some participants with clinical levels, given base rates of perinatal depression and other psychopathology). Note that some samples were recruited for other characteristics, such as prematurity or maternal history of childhood maltreatment. For the purposes of this meta-analysis, we coded these as community samples because they were not recruited specifically for elevated levels of maternal psychopathology. Clinical samples were defined as samples that comprised participants specifically recruited for clinical levels of maternal psychopathology or heightened levels of psychological distress. We coded studies comprising clinical and control groups as clinical, unless associations were provided separately for clinical and control groups, in which case we coded sample type at the level of the effect size.

***Mother Race and Ethnicity***

We extracted mother race and ethnicity in whatever way it was reported in each study. If mother race and ethnicity was not reported, we extracted infant race and ethnicity where possible. As this code was intended only to descriptively characterize the demographics of the included samples, we did not aim to code it in a way that provided consistent categorization of race and ethnicity across studies.

***Maternal Age***

We coded the mean and/or range of maternal age. Studies varied in when maternal age was assessed (e.g., at delivery, at study enrollment, at assessment), and in some studies it was not clear when maternal age was assessed. When maternal age at delivery was available, we coded this age. Otherwise, we used whatever maternal age information was available, with the rationale that all potential assessment time points were within a relatively small window (from pregnancy up to 2 years postnatal) and would still provide a general characterization of the age of the mothers included in the sample. We used this code only to characterize the demographics of the included samples.

***Parity***

We coded the percent of mothers who were primiparous (i.e., for whom the infant whose self-regulation was assessed was their first-born). If the article did not directly report primiparity of the mothers but did report the percentage of infants who were first children, we included this variable as an estimate of primiparity. We used this code only to characterize the demographics of the included samples.

***Country of Study***

For each study, we coded the country where data collection occurred. We used this code only to characterize the included samples.

***Maternal Depression Method***

We coded the method used to assess maternal depression as “self-report questionnaire” (the mother filled out a questionnaire about her own symptoms of depression), “other-report questionnaire” (someone other than the mother filled out a questionnaire about the mother’s symptoms of depression), “diagnostic interview” (an examiner interviewed the mother about her symptoms of depression and established the presence or absence of a depression diagnosis), “extracted from medical chart” (the researchers did not assess maternal depression directly but rather obtained depression information from medical chart records), “observational coding” (an examiner coded depressive symptoms based on observing the mother in some context), or “other” (the type of measure did not fit into any of the previously described categories).

***Maternal Depression Timing***

We coded the timing of maternal depression as “lifetime history” (assessed whether mothers had ever experienced depression), “pre-pregnancy” (assessed whether mothers had experienced depression at a time point prior to their pregnancy with the target child), “pregnancy” (assessed maternal depression occurring during pregnancy), “postnatal” (assessed maternal depression occurring after giving birth), or “other” (the timeframe assessed did not fit into any of the previously described categories or spanned multiple categories).

***Type of Depression Score***

We coded whether maternal depression scores were “binary,” “other categorical,” or “continuous.” Scores were coded as binary if the measure produced two categories (e.g., presence or absence of a diagnosis of depression; above or below a symptom-level cut-off). Scores were coded as other categorical if the measure produced more than two categories (e.g., low, moderate, and high levels of depression). In some cases, we included only two groups from multigroup studies (e.g., if a study included a control group, a group with depression, and a group with anxiety, we included only the control group and the group with depression). Scores were coded as continuous if they produced a continuous distribution of depression scores. When scores from a measure were presented in multiple ways in the article, we coded this variable according to whichever type of score was used in the relevant effect size.

***Reliability of Depression Measure***

We first coded the type(s) of reliability reported in a study (e.g., Cronbach’s $\alpha$, Cohen’s $\kappa$) and then coded the corresponding reliability value(s). Given that different studies reported different types of reliability that were not on comparable scales, for moderator analyses, we created a *post-hoc* binary reliability variable that indicated whether the reliability of a measure was above or below a threshold conventionally considered adequate for the given reliability metric. We used the following adequacy thresholds: Cronbach’s $\alpha$ ≥ .70 (Tavakol & Dennick, 2011), Cohen’s $\kappa$ ≥ .60 (McHugh, 2012), and intraclass correlation coefficient ≥ .75 (Koo & Li, 2016). All included effect sizes that reported reliability used depression measures with adequate reliability; thus, we did not include this variable in moderator analyses.

***Measure of Maternal Depression***

We coded the measure used to assess maternal depression for each effect size. We either extracted the name of the measure when an established measure was used or described the measurement when a combination of measures was used (e.g., if participants were first screened with a questionnaire and then assessed with a diagnostic interview). We used this code only to describe the maternal depression measures used in the included studies.

***Self-Regulation Method***

We coded the method used to assess infant self-regulation as “questionnaire” (a reporter, typically the mother, filled out a questionnaire about the infant’s self-regulation; e.g., Infant Behavior Questionnaire-Revised, Gartstein & Rothbart, 2003), “observational coding” (an examiner coded infant self-regulation based on observing the infant engaging in a task; e.g., behavioral coding during the Still Face Paradigm), “clinician assessment” (an examiner performed a standardized clinical protocol with the infant and measured responses; e.g., Neonatal Behavioral Assessment Scale, Brazelton & Nugent, 2011), “interview” (an examiner interviewed a reporter, typically the mother, about the infant’s self-regulation; e.g., Baby-DIPS, Schneider, S., & Wolke, D., 2007), “eye-tracking task” (the infant’s gaze patterns were assessed with eye tracking technology during a task; e.g., eye tracking during a selective attention task), and “other” (the type of measure did not fit into any of the previously described categories).

***Reliability of Self-Regulation Measure***

We first coded the type(s) of reliability reported in a study and then coded the corresponding reliability value(s). For moderator analyses, we created a *post-hoc* binary reliability variable that indicated whether the reliability of a measure was above or below a threshold conventionally considered adequate for the given reliability metric. We used the following adequacy thresholds: Cronbach’s $\alpha$ and McDonald’s $\omega$≥ .70 (Tavakol & Dennick, 2011), Cohen’s $\kappa$ ≥ .60 (McHugh, 2012), Intraclass Correlation Coefficient ≥ .75 (Koo & Li, 2016), and percent rater agreement ≥ 70.

***Independence of Self-Regulation Measure***

With this code, which we created *post-hoc*, given the importance of caregiver contributions to early self-regulation, we sought to characterize the extent to which each measure of self-regulation captured regulatory strategies employed independently by the infant or more co-regulatory processes. We coded a measure as “independent” if it comprised regulatory strategies employed by the infant without clear maternal contributions. We coded a measure as “bids to mother” if it comprised strategies employed by the infant to engage maternal regulatory support. We coded a measure as “combined” if it comprised some combination of a) independent infant strategies, b) bids for maternal assistance, and c) an index of infant responsiveness to maternal regulatory support*.* Finally, we included a code (“distress”) to capture measures that did not assess regulation directly but rather conceptualized infant distress as an index of regulatory failure.

***Trait- or Process-Oriented Measure of Self-Regulation***

With this code, which was also created *post-hoc,* we aimed to capture whether self-regulation was conceptualized as a trait-like aspect of infant temperament or as a process deployed in real time to regulate the level of reactivity. We coded a measure as “trait” if it captured an infant’s general level of regulation. We coded a measure as “process” if it documented specific regulatory strategies used (or failure to apply regulatory strategies, i.e., distress) at a specific moment, typically the time of assessment.

***Context of Self-Regulation Assessment***

With this code, we aimed to capture whether regulation was assessed in the context of an acute stressor or not. We coded an effect size as “stressor” if it was assessed during a task intended to be stressful. We coded all other effect sizes as “not stressor.”

***Self-Regulation-Related Construct Assessed***

We coded which construct (i.e., self-regulation or one of the related constructs conceptualized as falling under the umbrella of self-regulation) was assessed for each effect size. We deferred to whatever terminology the authors of a given study used to categorize the type of construct (e.g., if the authors described a measure as assessing executive function, we coded that construct as “executive function”). We used this code only to describe the specific constructs assessed in the included studies.

***Measure of Self-Regulation***

We coded the specific measure that was used to assess infant self-regulation for each effect size. We either extracted the name of the measure when an established measure was used or described the measurement when only a description of the measure was provided. We used this code only to describe the self-regulation measures used in the included studies.

**Supplemental Tables**

**Table S1**

*Conceptualization of Infant Self-Regulation Moderators*

| Moderator | Levels | Definitions | Example(s) |
| --- | --- | --- | --- |
| Independence | Independent | Assessed self-regulatory strategies employed by the infant without clear maternal contributions | Gaze aversion coded during a stressful stimulus; state regulation scores on the NBAS |
|  | Bids to Mother | Assessed self-regulatory strategies employed by the infant to engage maternal regulatory support | Looks to mother coded during a stressful stimulus |
|  | Combined | Assessed some combination of independent infant strategies, bids for maternal assistance, and an index of infant responsiveness to maternal regulatory support | Orienting/Regulatory Capacity factor of the IBQ-R (which includes items assessing independent processes like duration of orienting as well as responses to maternal input such as soothability) |
|  | Distress | Did not assess self-regulation directly but rather conceptualized infant distress as an index of self-regulatory failure | Negative Affectivity factor of the IBQ-R; negative affect coded during a stressful stimulus |
| Trait- vs Process-Oriented | Trait | Assessed an infant’s general level of self-regulation | Orienting/Regulatory Capacity factor of the IBQ-R |
|  | Process | Assessed specific self-regulatory strategies used (or failure to apply regulatory strategies) at a specific moment | Gaze aversion coded during a stressful stimulus |
| Context | Stressor | Assessed self-regulation in the context of an acute stressor | Self-regulatory behaviors coded during the Still Face Paradigm |
|  | Not Stressor | Assessed self-regulation outside of an acute stressor or in general (i.e., typical responses across various daily-life contexts) | Self-regulatory behaviors coded during a mother-infant free play task; Orienting/Regulatory Capacity factor of the IBQ-R |

*Note.* IBQ-R = Infant Behavior Questionnaire-Revised; NBAS = Neonatal Behavioral Assessment Scale.

**Table S2**

*Descriptive Statistics of Study Variables and Potential Moderators*

| Variable | *k* | *M (SD),* Range | *n* (%) |
| --- | --- | --- | --- |
| **Study Information and Sample Characteristics** | | | |
| Descriptive Variables |  |  |  |
| Maternal Age (years) | 51^a^ | 29.87 (2.55), 15 – 51 |  |
| Parity (% primiparous) | 26^a^ | 65.27 (28.05), 18 – 100 |  |
| Country of Study | 61^a^ |  |  |
| United States |  |  | 33 (54.1) |
| Canada |  |  | 5 (8.2) |
| Germany |  |  | 3 (4.9) |
| Portugal |  |  | 3 (4.9) |
| United Kingdom |  |  | 3 (4.9) |
| Finland |  |  | 2 (3.3) |
| Italy |  |  | 2 (3.3) |
| Australia |  |  | 1 (1.6) |
| Brazil |  |  | 1 (1.6) |
| Denmark |  |  | 1 (1.6) |
| France |  |  | 1 (1.6) |
| Israel |  |  | 1 (1.6) |
| Japan |  |  | 1 (1.6) |
| Palestine |  |  | 1 (1.6) |
| Poland |  |  | 1 (1.6) |
| South Africa |  |  | 1 (1.6) |
| Other^b^ |  |  | 1 (1.6) |
| Potential Moderators |  |  |  |
| Infant Sex (% female) | 52^a^ | 49.79 (5.03), 40 – 63 |  |
| Infant Age (months) | 188 | 6.74 (3.86), 0.00 – 16.10 |  |
| Type of Sample | 193 |  |  |
| Community |  |  | 139 (72.0) |
| Clinical |  |  | 54 (28.0) |
| **Maternal Depression Information** | | | |
| Potential Moderators |  |  |  |
| Maternal Depression Method | 193 |  |  |
| Self-Report Questionnaire |  |  | 156 (80.8) |
| Diagnostic Interview |  |  | 30 (15.5) |
| Medical Chart |  |  | 3 (1.6) |
| Other^c^ |  |  | 4 (2.1) |
| Maternal Depression Timing | 193 |  |  |
| Postnatal |  |  | 109 (56.5) |
| Pregnancy |  |  | 72 (37.3) |
| Lifetime History |  |  | 1 (0.5) |
| Other^d^ |  |  | 11 (5.7) |
| Maternal Depression Score Type | 193 |  |  |
| Continuous |  |  | 150 (77.7) |
| Binary |  |  | 43 (22.3) |
| Reliability of Maternal Depression Measure | 106 |  |  |
| Adequate |  |  | 106 (100.0) |
| **Infant Self-Regulation Information** | | | |
| Descriptive Variables |  |  |  |
| Construct Assessed^e^ | 193 |  |  |
| Emotion(al) Regulation^f^ |  |  | 37 (19.2) |
| Self-Regulation |  |  | 37 (19.2) |
| Regulation |  |  | 34 (17.6) |
| Effortful Control |  |  | 29 (15.0) |
| Stress Regulation |  |  | 24 (12.4) |
| Mutual Regulation^g^ |  |  | 13 (6.7) |
| State Regulation |  |  | 6 (3.1) |
| Attention(al) Control |  |  | 4 (2.1) |
| Attention Regulation |  |  | 4 (2.1) |
| Behavior(al) Regulation |  |  | 3 (1.6) |
| Multiple^h^ |  |  | 2 (1.0) |
| Potential Moderators |  |  |  |
| Self-Regulation Method | 193 |  |  |
| Questionnaire |  |  | 104 (53.9) |
| Observational Coding of Task |  |  | 66 (34.2) |
| Clinician Assessment |  |  | 18 (9.3) |
| Eye Tracking |  |  | 4 (2.1) |
| Interview |  |  | 1 (0.5) |
| Reliability of Self-Regulation Measure | 93 |  |  |
| Adequate |  |  | 71 (76.3) |
| Inadequate |  |  | 22 (23.7) |
| Independence of Self-Regulation | 193 |  |  |
| Combined |  |  | 80 (41.5) |
| Independent |  |  | 79 (40.9) |
| Distress |  |  | 29 (15.0) |
| Bids to Mother |  |  | 5 (2.6) |
| Trait/Process | 193 |  |  |
| Trait |  |  | 107 (55.4) |
| Process |  |  | 86 (44.6) |
| Context of Self-Regulation Assessment | 193 |  |  |
| Not Stressor |  |  | 156 (80.8) |
| Stressor |  |  | 37 (19.2) |

^a^ These codes did not vary within studies; thus, they were collected only at the level of study and had a total possible *k* of 61. The total possible *k* for all other codes was 193.

^b^ Data for this study were collected online with no country restrictions.

^c^ These four effect sizes used a self-report questionnaire for initial screening followed by diagnostic interview.

^d^ For these effects sizes, maternal depression spanned both the pregnancy and postnatal periods (e.g., an average across both periods or a measure asking mothers to report about both periods).

^e^ Any constructs listed in Table 1 that do not appear here were not assessed in any of the included records.

^f^ Or affect(ive) regulation, mood regulation, or regulation of a specific emotion (e.g., fear regulation).

^g^ Or co- or dyadic regulation.

^h^ The self-regulation measure used in these two effect sizes was labeled by the study authors as capturing a combination of two different self-regulation related constructs (inhibition and updating).

**Supplemental References**

Appelbaum, M., Cooper, H., Kline, R. B., Mayo-Wilson, E., Nezu, A. M., & Rao, S. M. (2018). Journal article reporting standards for quantitative research in psychology: The APA Publications and Communications Board task force report. *American Psychologist*, *73*(1), 3–25. https://doi.org/10.1037/amp0000191

Brazelton, T.B., and Nugent, J.K. (2011). *The Neonatal Behavioral Assessment Scale*. Mac Keith Press.

Gartstein, M. A., & Rothbart, M. K. (2003). Studying infant temperament via the Revised Infant Behavior Questionnaire. *Infant Behavior and Development, 26* (1), 64-86.

Higgins J, Morgan R, Rooney A, Taylor K, Thayer K, Silva R, Lemeris C, Akl A, Arroyave W, Bateson T, Berkman N, Demers P, Forastiere F, Glenn B, Hróbjartsson A, Kirrane E, LaKind J, Luben T, Lunn R, McAleenan A, … & Sterne J. Risk Of Bias In Non- randomized Studies - of Exposure (ROBINS-E). Launch version, 20 June 2023. Available from: <https://www.riskofbias.info/welcome/robins-e-tool>.

Koo, T. K., & Li, M. Y. (2016). A guideline of selecting and reporting intraclass correlation coefficients for reliability research. *Journal of Chiropractic Medicine*, *15*(2), 155–163. https://doi.org/10.1016/j.jcm.2016.02.012

McHugh, M. L. (2012). Interrater reliability: The kappa statistic. *Biochemia Medica*, *22*(3), 276–282.

Page MJ, McKenzie JE, Bossuyt PM, Boutron I, Hoffmann TC, Mulrow CD, et al. The PRISMA 2020 statement: an updated guideline for reporting systematic reviews. BMJ 2021;372:n71. doi: 10.1136/bmj.n71

Schneider, S., & Wolke, D. (2007). Diagnostisches Interview zur Erfassung von Regulationsstörungen im Säuglings-und Kleinkindalter (Baby-DIPS). *Universität Basel, Basel*.

R Core Team (2021). R: A language and environment for statistical computing. R Foundation for Statistical Computing, Vienna, Austria. https://www.R-project.org/.

Tavakol, M., & Dennick, R. (2011). Making sense of Cronbach’s alpha. *International Journal of Medical Education*, *2*, 53–55. <https://doi.org/10.5116/ijme.4dfb.8dfd>

Viechtbauer, W. (2010). Conducting Meta-Analyses in R with the metafor Package. *Journal of Statistical Software*, *36*(3), 1–48. <https://doi.org/10.18637/jss.v036.i03>

**Appendix A**

**Table A1**

*Summary of Maternal Depression and Infant Self-Regulation Characteristics and Effect Size Statistics for Associations between Maternal Perinatal Depression and Infant Self-Regulation*

|  |  |  |  |  | Maternal Depression Information | | | Infant Self-Regulation Information | | | |  |  |
| --- | --- | --- | --- | --- | --- | --- | --- | --- | --- | --- | --- | --- | --- |
| Study | Age | Sex | Sam | Maternal  Race/Ethnicity | Tim | Measure  (Reliability) | Met | Con | Measure (Reliability) | Met | | *r*^a^ | *N* |
| Barona et al., 2017 | 0.29 | - | Cl | 81% White,  9% Other | Pre | BDI (-) | SRQ | Neur | NBAS Reg (-) | CA | | .04^b^ | 64 |
| Bates et al., 2020 | 11.18 | 56 | Com | 52% Black (16% of whom were mixed race),  48% Other (94% of whom were White only, 6% Asian only, 5% American Indian/Alaskan Native only),  9% Latino/Hispanic,  5% Unknown^c^ | Pos | EPDS (.85) | SRQ | SR, EC | IBQ-R-VSF ORC (.80) | Q | | -.18 | 129 |
| Beckwith, 2014 | 0.02 | 47 | Cl | 83% African American, 14% Hispanic, 1% Caucasian, <1% Asian, 2% Other | Pre | BDI-II (-) | SRQ | SR | NBAS SR (-) | CA | | .00 | 71 |
|  | 0.02 |  | Cl |  | Pre | BDI-II (-) | SRQ | SR | NBAS SR (-) | CA | | -.09 | 71 |
|  | 1.00 |  | Cl |  | Pre | BDI-II (-) | SRQ | SR | NBAS SR (-) | CA | | .09 | 66 |
|  | 1.00 |  | Cl |  | Pre | BDI-II (-) | SRQ | SR | NBAS SR (-) | CA | | .00 | 66 |
| Bosquet Enlow et al., 2011 | 6.89 | 46 | Com | 52% White,  42% Black,  25% Hispanic,  4% Asian,  2% Multiracial | Pos | EPDS  (.91) | SRQ | ER | IBQ-R  FR  (.84) | Q | | -.32 | 52 |
|  | 14.07 |  | Com |  | Pos | EPDS  (.91) | SRQ | Reg | ITSEA  Dysreg (.91) | Q | | -.31 | 52 |
| Buthmann, 2021 | 6.96 | 50 | Com | 50 % Hispanic,  24% Black,  15% White,  10% Asian,  2% Other | Pre | EPDS (.74) | SRQ | ER | IBQ-R  ORC (-) | Q | | -.15 | 360 |
|  | 6.96 |  | Com |  | Pos | EPDS (.79) | SRQ | ER | IBQ-R  ORC (-) | Q | | -.22 | 360 |
| Camerota et al., 2023 | 0.00 | 46 | Com | 51% White, 25% Black, 16% Hispanic, 6% Asian, 8% Other Race or Ethnicity | Oth | Diagnosed pre- or postnatally via self-report or medical chart (-) | MC | Reg | Hypo-aroused dysreg NNNS profile (-) | CA | | -.10 | 732 |
|  | 0.00 |  | Com |  | Oth | Diagnosed pre- or postnatally via self-report or medical chart (-) | MC | Reg | Hyper-aroused dysreg NNNS profile (-) | CA | | -.24 | 850 |
| Camerota, 2018 | 3.56 | 46 | Com | 100% African American | Pos | CESD (.89) | SRQ | Reg | IBQ-R ORC (.76) | Q | | -.10 | 76 |
|  | 6.47 |  | Com |  | Pos | CESD (.89) | SRQ | AR | Looking time during Puppet Task (-) | Obs | | .24 | 72 |
| Capelli et al., 2023 | 3.00 | 50 | Com | - | Pos | BDI-II (.88) | SRQ | Reg | IBQ-R ORC (.86) | Q | | -.21 | 261 |
|  | 6.00 |  | Com |  | Pos | BDI-II (.90) | SRQ | Reg | IBQ-R ORC (.88) | Q | | -.24 | 217 |
| Choe et al., 2020^d^ | 7.00 | 53 | Com | 74% Caucasian,  13% African American,  12% Biracial or Other,  7% Not Reported^c^ | Pos | CESD (.88) | SRQ | Reg | CPQ (.81) | Q | | -.12 | 252 |
| Choe et al., 2013^d^ | 7.00 | 53 | Com | 72% Caucasian,  14% Biracial or Other, 12% African American | Pos | CESD (.88) | SRQ | Reg | CPQ, SHS, and FPS composite  (.50) | Q | | -.24 | 251 |
| Colaizzi, 2013 | 3.00 | 62 | Com | 87% Caucasian | Pos | SCL-90-R Dep (-) | SRQ | ER | Response to infant crying task (-) | Obs | | -.12 | 29 |
|  | 6.00 |  | Com |  | Pos | SCL-90-R Dep (-) | SRQ | ER | Response to infant crying task (-) | Obs | | -.26 | 31 |
|  | 9.00 |  | Com |  | Pos | SCL-90-R Dep (-) | SRQ | ER | Response to infant crying task (-) | Obs | | -.23 | 23 |
| Conradt et al., 2013 | *-* | 53 | Com | 72% Caucasian,  17% African American,  11% Other,  <1% Unknown | Pre | Structured chart review (-) | MC | SR | NNNS  SR  (-) | CA | | .05 | 461 |
| Dias et al., 2023 | 0.50 | 50 | Com | 91% White, 9% Other | Pre | EPDS (.86^e^) | SRQ | Reg | IBQ-R ORC (.59^e^) | Q | | .02 | 172 |
| Eskola et al., 2021^f^ | 3.00 | 53 | Cl | - | Pos | EPDS (-) | SRQ | BR | RPQ (-) | Q | | -.24 | 176 |
| Evans, 2020 | 4.75 | 63 | Com | 92% White British/White | Pos | EPDS (.90) | SRQ | Reg, EC | IBQ-R-VSF ORC (.82) | Q | | -.35 | 137 |
| Fantini, 2015 | 0.20 | - | Com | 66% White, 34% Black and Minority | Pre | SCID-IV (-) | DI | State | NBAS State  (-) | CA | | -.26 | 114 |
| Foss et al., 2023 | 6.70 | 48 | Com | 44% Black, 35% Hispanic/Latina, 17% White, 5% Other | Pre | EPDS (-) | SRQ | Str | IBQ-R ORC (-) | Q | | -.03 | 677 |
|  | 6.70 |  | Com |  | Pre | EPDS (-) | SRQ | Str | SFP Recovery 1 NM (-) | Obs | | .04 | 412 |
|  | 6.70 |  | Com |  | Pre | EPDS (-) | SRQ | Str | SFP Recovery 2 NM (-) | Obs | | .01 | 412 |
| Frigerio & Molteni, 2022 | 2.95 | 48 | Com | - | Pre | EPDS (-) | SRQ | ER | IBQ-VSF NE (-) | Q | | -.28 | 108 |
|  | 2.95 |  | Com |  | Pos | EPDS (-) | SRQ | ER | IBQ-VSF NE (-) | Q | | -.18 | 106 |
| Garcia et al., 2022 | 9.50 | 57 | Cl | - | Pre | EPDS (-) | SRQ | EC | IBQ-R ORC (-) | Q | | .12 | 34 |
|  | 9.50 |  | Cl |  | Pos | EPDS (-) | SRQ | EC | IBQ-R ORC (-) | Q | | -.26 | 35 |
|  | 9.50 |  | Cl |  | Oth | EPDS (-) | SRQ | EC | IBQ-R ORC (-) | Q | | -.35 | 39 |
|  | 9.50 |  | Cl |  | Pre | EPDS (-) | SRQ | EC | IBQ-R Sooth (-) | Q | | -.01 | 34 |
|  | 9.50 |  | Cl |  | Pos | EPDS (-) | SRQ | EC | IBQ-R Sooth (-) | Q | | -.42 | 35 |
|  | 9.50 |  | Cl |  | Oth | EPDS (-) | SRQ | EC | IBQ-R Sooth (-) | Q | | -.22 | 39 |
|  | 9.50 |  | Cl |  | Pre | EPDS (-) | SRQ | EC | IBQ-R LIP (-) | Q | | .02 | 34 |
|  | 9.50 |  | Cl |  | Pos | EPDS (-) | SRQ | EC | IBQ-R LIP (-) | Q | | -.05 | 35 |
|  | 9.50 |  | Cl |  | Oth | EPDS (-) | SRQ | EC | IBQ-R LIP (-) | Q | | -.28 | 39 |
|  | 9.50 |  | Cl |  | Pre | EPDS (-) | SRQ | EC | IBQ-R Cuddliness (-) | Q | | .08 | 34 |
|  | 9.50 |  | Cl |  | Pos | EPDS (-) | SRQ | EC | IBQ-R Cuddliness (-) | Q | | -.27 | 35 |
|  | 9.50 |  | Cl |  | Oth | EPDS (-) | SRQ | EC | IBQ-R Cuddliness (-) | Q | | -.41 | 39 |
|  | 9.50 |  | Cl |  | Pre | EPDS (-) | SRQ | EC | IBQ-R DO (-) | Q | | .17 | 34 |
|  | 9.50 |  | Cl |  | Pos | EPDS (-) | SRQ | EC | IBQ-R DO (-) | Q | | .03 | 35 |
|  | 9.50 |  | Cl |  | Oth | EPDS (-) | SRQ | EC | IBQ-R DO (-) | Q | | -.21 | 39 |
|  | 9.50 |  | Cl |  | Pre | EPDS (-) | SRQ | EC | IBQ-R DVR (-) | Q | | .05 | 34 |
|  | 9.50 |  | Cl |  | Pos | EPDS (-) | SRQ | EC | IBQ-R DVR (-) | Q | | -.34 | 35 |
|  | 9.50 |  | Cl |  | Oth | EPDS (-) | SRQ | EC | IBQ-R DVR (-) | Q | | -.25 | 39 |
| Gartstein & Hancock, 2019 | 4.03 | 49 | Com | 92 % Caucasian, 4% African American, 3% Asian, 2% Hispanic/Latino | Pos | BDI-II (.89) | SRQ | Reg | IBQ-R ORC (.65) | Q | | -.02 | 131 |
|  | 6.08 |  | Com |  | Pos | BDI-II (.89) | SRQ | Reg | IBQ-R ORC (.65) | Q | | .02 | 113 |
|  | 7.97 |  | Com |  | Pos | BDI-II (.89) | SRQ | Reg | IBQ-R ORC (.65) | Q | | .13 | 102 |
|  | 10.03 |  | Com |  | Pos | BDI-II (.89) | SRQ | Reg | IBQ-R ORC (.65) | Q | | -.07 | 86 |
|  | 12.04 |  | Com |  | Pos | BDI-II (.89) | SRQ | Reg | IBQ-R ORC (.65) | Q | | .06 | 97 |
| Georg et al., 2021 | 8.57 | 44 | Com | 86% German origin | Pos | SCL-90-R Dep (.86) | SRQ | Reg | CFS (.81) | Q | | -.24 | 154 |
| Gerardin et al., 2011 | 0.10 | - | Cl | *-* | Pre | MADR, MINI (-) | Oth | State | NBAS State  (-) | CA | | -.27 | 54 |
|  | 12.00 |  | Cl |  | Pre | MADR, MINI (-) | Oth | Reg | ITSEA NE (-) | CA | | -.20 | 75 |
|  | 12.00 |  | Cl |  | Pre | MADR, MINI (-) | Oth | Reg | ITSEA TS (-) | CA | | -.17 | 75 |
|  |  |  |  |  |  |  |  |  |  |  | |  |  |
| Goodman et al., 2017 | 3.00 | 54 | Cl | 68% African American,  32% European American | Pre | BDI-II  (.75) | SRQ | Reg | IBQ-R  ORC  (-) | Q | | .16 | 76 |
| Granat et al., 2017 | 9.00 | 56 | Cl | - | Pos | SCID-IV-R  (>85^g^) | DI | SR | SR in Joy-Mother Task  (.82^h^) | Obs | | .24 | 81 |
|  | 9.00 |  | Cl |  | Pos | SCID-IV-R  (>85^g^) | DI | SR | SR in Anger-Mother Task  (.82^h^) | Obs | | .02 | 81 |
|  | 9.00 |  | Cl |  | Pos | SCID-IV-R  (>85^g^) | DI | SR | SR in Anger-Stranger Task  (.82^h^) | Obs | | .03 | 81 |
| Gustafsson et al., 2016 | 4.00 | 50 | Com | 63% Latina | Pre | HRSD (-) | DI | Reg | IBQ-R ORC  (.90) | Q | | -.09 | 48 |
| Gustafsson et al., 2018 | 6.80 | 43 | Com | 77% Non-Hispanic Caucasian, 7% Asian or Southeast Asian, 5% African American,  5% Hispanic, 3% Biracial, 3% Native American | Pre | CESD  (.93) | SRQ | ER | SFP and AR escape (.50^h^) | Obs | | .11 | 42 |
|  | 6.80 |  | Com |  | Pos | CESD  (.94) | SRQ | ER | SFP and AR escape (.50^h^) | Obs | | .23 | 42 |
|  | 6.80 |  | Com |  | Pre | CESD  (.93) | SRQ | ER | SFP and AR attention seeking(.50^h^) | Obs | | -.33 | 42 |
|  | 6.80 |  | Com |  | Pos | CESD  (.94) | SRQ | ER | SFP and AR attention seeking (.50^h^) | Obs | | -.10 | 42 |
|  | 6.80 |  | Com |  | Pre | CESD  (.93) | SRQ | ER | IBQ-R  Sooth (.82) | Q | | -.15 | 42 |
|  | 6.80 |  | Com |  | Pos | CESD  (.94) | SRQ | ER | IBQ-R  Sooth (.82) | Q | | -.19 | 42 |
| Halligan et al., 2013 | - | 52 | Cl | 86% Caucasian | Pos | EPDS (.82) | SRQ | ER, State | NBAS State  (.66) | CA | | -.28 | 121 |
|  | - |  | Cl |  | Pos | EPDS (.82) | SRQ | ER | BSID and LabTAB ER composite (.87) | CA | | .01 | 105 |
| Hart & Behrens, 2013 | 11.10 | 56 | Com | 81% European American, 13% Latina,  3% African American,  3% Asian | Pos | CESD (.88) | SRQ | Fear | LabTAB fear  (.81^h^) | Obs | | -.18 | 84 |
| Hunter et al., 2021 | 3.00 | 48 | Com | - | Pre | CESD (-) | SRQ | SR | IBQ-R ORC (-) | Q | | -.17 | 127 |
|  | 3.00 |  | Com |  | Pre | SCID-IV (-) | DI | SR | IBQ-R ORC (-) | Q | | -.10 | 137 |
| Isosävi et al., 2017 | 4.00 | - | Com | - | Pre | EPDS (.78) | SRQ | Str | IBQ-R DL (-) | Q | | .07 | 477 |
|  | 4.00 |  | Com |  | Pre | EPDS (.78) | SRQ | Str | IBQ-R DL (-) | Q | | .03 | 477 |
|  | 4.00 |  | Com |  | Pre | EPDS (.78) | SRQ | Str | IBQ-R DL (-) | Q | | .05 | 477 |
|  | 4.00 |  | Com |  | Pre | EPDS (.78) | SRQ | Str | IBQ-R FR (-) | Q | | .08 | 477 |
|  | 4.00 |  | Com |  | Pre | EPDS (.78) | SRQ | Str | IBQ-R FR (-) | Q | | -.01 | 477 |
|  | 4.00 |  | Com |  | Pre | EPDS (.78) | SRQ | Str | IBQ-R FR (-) | Q | | -.02 | 477 |
|  | 4.00 |  | Com |  | Pre | EPDS (.78) | SRQ | Str | IBQ-R Fear  (-) | Q | | .04 | 477 |
|  | 4.00 |  | Com |  | Pre | EPDS (.78) | SRQ | Str | IBQ-R Fear  (-) | Q | | .01 | 477 |
|  | 4.00 |  | Com |  | Pre | EPDS (.78) | SRQ | Str | IBQ-R Fear  (-) | Q | | .00 | 477 |
|  | 4.00 |  | Com |  | Pre | EPDS (.78) | SRQ | Str | IBQ-R Sadness (-) | Q | | .07 | 477 |
|  | 4.00 |  | Com |  | Pre | EPDS (.78) | SRQ | Str | IBQ-R  Sadness (-) | Q | | .06 | 477 |
|  | 4.00 |  | Com |  | Pre | EPDS (.78) | SRQ | Str | IBQ-R  Sadness (-) | Q | | .03 | 477 |
|  | 4.00 |  | Com |  | Pre | EPDS (.78) | SRQ | Str | IBQ-R Sooth 1 Parcel (-) | Q | | .06 | 477 |
|  | 4.00 |  | Com |  | Pre | EPDS (.78) | SRQ | Str | IBQ-R Sooth 1 Parcel (-) | Q | | .05 | 477 |
|  | 4.00 |  | Com |  | Pre | EPDS (.78) | SRQ | Str | IBQ-R Sooth 1 Parcel (-) | Q | | .03 | 477 |
|  | 4.00 |  | Com |  | Pre | EPDS (.78) | SRQ | Str | IBQ-R Sooth 2 Parcel (-) | Q | | .05 | 477 |
|  | 4.00 |  | Com |  | Pre | EPDS (.78) | SRQ | Str | IBQ-R Sooth 2 Parcel (-) | Q | | .00 | 477 |
|  | 4.00 |  | Com |  | Pre | EPDS (.78) | SRQ | Str | IBQ-R Sooth 2 Parcel (-) | Q | | .03 | 477 |
|  | 4.00 |  | Com |  | Pre | EPDS (.78) | SRQ | Str | IBQ-R Sooth 3 Parcel (-) | Q | | .02 | 477 |
|  | 4.00 |  | Com |  | Pre | EPDS (.78) | SRQ | Str | IBQ-R Sooth 3 Parcel (-) | Q | | .04 | 477 |
|  | 4.00 |  | Com |  | Pre | EPDS (.78) | SRQ | Str | IBQ-R Sooth 3 Parcel (-) | Q | | .01 | 477 |
| Jia, 2014 | 6.42 | 48 | Com | 88% White, 4% African American, 4% Asian, 4% Hispanic | Pos | CESD (.89) | SRQ | EC | IBQ-R-VSF ORC (mother-reported; .77) | Q | | -.16 | 76 |
|  | 6.42 |  | Com |  | Pos | CESD (.91) | SRQ | EC | IBQ-R-VSF ORC (mother-reported; .77) | Q | | -.17 | 75 |
|  | 6.42 |  | Com |  | Pos | CESD (.89) | SRQ | EC | IBQ-R-VSF ORC (father-reported; .73) | Q | | .11 | 75 |
|  | 6.42 |  | Com |  | Pos | CESD (.91) | SRQ | EC | IBQ-R-VSF ORC (father-reported; .73) | Q | | -.05 | 75 |
| Jones, 2012 | 0.05 | 44 | Cl | 70% Caucasian, 18% African American, 7% Hispanic,  5% Mixed Racial Groups | Oth | CESD, DIS  (-) | Oth | State | NBAS  State (.90^h^) | CA | | -.18 | 101 |
| Kajanoja et al., 2022^f^ | 6.00 | 54 | Com | - | Pre | EPDS (.82) | SRQ | SR | IBQ-R ORC (.83) | Q | -.10^b^ | | 1173 |
| Kelsey et al., 2021 | 0.84 | 43 | Com | 67% White, 19% Black, 4% Pacific Islander, 4% South Asian, 3% Asian, 4% Other ^c^ | Pos | EPDS (-) | SRQ | Reg | IBQ-R-SF ORC (.78) | Q | .00 | | 75 |
| Khoury et al., 2016 | 15.41 | 52 | Com | 72% White, 10% Asian, 4% African Canadian, 14% Other Ethnicities | Pos | BDI-II  (.89) | SRQ | ER | Reg in Toy Frustration  (.83^d^) | Obs | | .15 | 165 |
| Krzeczkowski et al., 2021 | 5.75 | 60 | Cl | - | Oth | Structured DI (-) | DI | ER | IBQ-R ORC (.88) | Q | | -.25 | 79 |
| Kusangi et al., 2014 | 2.90 | 49 | Com | - | Pos | ZSRD (.86) | SRQ | Reg | IBQ-R ORC (.66) | Q | | -.18 | 55 |
|  | 10.70 | 51 | Com |  | Pos | ZSRD (.86) | SRQ | Reg | IBQ-R ORC (.59) | Q | | -.07 | 51 |
|  | 16.10 | 50 | Com |  | Pos | ZSRD (.86) | SRQ | Reg | IBQ-R ORC (.38) | Q | | -.16 | 52 |
| Longoria, 2015 | 11.80 | 50 | Com | 90% Caucasian, 8% Asian,  3% Biracial | Pos | CESD  (.83) | SRQ | SR | ASQ-SE  SR (.49) | Q | | -.16^b^ | 40 |
| Luecken et al., 2019^i^ | 3.00 | *-* | Com | 100% Mexican or Mexican American | Pre | EPDS  (.86) | SRQ | Dyadic Reg | Dyadic Dysreg in various tasks  (70^g^) | Obs | | -.11 | 204 |
| Martinez-Torteya et al., 2014^j^ | 6.14 | 48 | Com | 56% Caucasian, 22% African American,  13% Multiracial,  5% Other,  4% Hispanic | Pos | PDSS (-) | SRQ | BR | SFP PR (.86) | Obs | | .07 | 153 |
| Martinez-Torteya et al., 2015^j^ | 7.00 | 44 | Com | 63% Caucasian; 24% African American; 11% Asian/Pacific Islander, Latina, or Biracial | Pos | PDSS  (.96) | SRQ | Reg | IBQ-R  ORC (.88) | Q | | .09 | 167 |
| Martini et al., 2017 | *-* | 49 | Com | - | LTH | CIDI (-) | DI | Reg | Baby-DIPS Excessive Infant Crying (-) | Int | | .05 | 286 |
| Mattera et al., 2022 | 2.33 | 55 | Com | 81% White, 7% Hispanic or Latina, 5% Asian American, 2% American Indian/Alaska Native, 2% Native Hawaiian or Pacific Islander, 3.4% Other | Pre | EPDS (.84) | SRQ | SR | IBQ-R ORC (.80) | Q | | -.25 | 40 |
|  | 2.33 |  | Com |  | Pre | EPDS (.84) | SRQ | SR | IBQ-R DO (.80) | Q | | .10 | 41 |
|  | 2.33 |  | Com |  | Pre | EPDS (.84) | SRQ | SR | IBQ-R Sooth (.80) | Q | | -.24 | 45 |
|  | 2.33 |  | Com |  | Pre | EPDS (.84) | SRQ | SR | IBQ-R LIP (.80) | Q | | -.27 | 43 |
|  | 2.33 |  | Com |  | Pre | EPDS (.84) | SRQ | SR | IBQ-R Cuddliness (.80) | Q | | -.21 | 45 |
| Menke, 2014^j^ | 6.00 | *-* | Cl | 65% Caucasian | Pos | PDSS (.96) | SRQ | ER | SFP NA and Sooth (.93^k^) | Obs | | -.16 | 71 |
| Miller-Graff & Scheid, 2020 | 1.79 | - | Com | 39% Black or African American,  37% White or Caucasian, 20% Hispanic or Latina, 5% Biracial or Multiracial | Pre | CESD  (.89) | SRQ | Reg | IBQ-R-VSF  ORC (.63) | Q | | -.01 | 69 |
| Morris & Saxbe, 2023 | 3.36 | - | Com | 73% White, 11% Hispanic/Latinx, 7% Black, 7% Asian/Pacific Islander, 2% Multiracial/Other, <1% American Indian/Alaska Native | Pre | BDI-II (-) | SRQ | EC | IBQ-R ORC (-) | Q | | -.07 | 335 |
|  | 3.36 |  | Com |  | Pos | BDI-II (-) | SRQ | EC | IBQ-R ORC (-) | Q | | -.10 | 335 |
| Niedzwiecka et al., 2018 | 5.47 | 51 | Com | 100% Caucasian | Pos | EPDS (-) | SRQ | AC | Gap Effect from Gap-and-Overlap Task (-) | ET | | .04 | 55 |
|  | 11.59 |  | Com |  | Pos | EPDS (-) | SRQ | AC | Gap Effect from Gap-and-Overlap Task (-) | ET | | -.17 | 55 |
|  | 5.47 |  | Com |  | Pos | EPDS (-) | SRQ | AC | Overlap Effect from Gap-and-Overlap Task  (-) | ET | | -.05 | 55 |
|  | 11.59 |  | Com |  | Pos | EPDS (-) | SRQ | AC | Overlap Effect from Gap-and-Overlap Task  (-) | ET | | .00 | 55 |
|  | 5.47 |  | Com |  | Pos | EPDS (-) | SRQ | Reg | IBQ-R-VSF ORC  (-) | Q | | -.09 | 55 |
| Nolvi et al., 2018^f^ | 8.10 | - | Cl | - | Pre | EPDS  (.84) | SRQ | EF, Inh, Up | Modified AB/Delayed Response Task (81^e^) | Obs | | -.08 | 214 |
|  | 8.10 |  | Cl |  | Pos | EPDS  (.84) | SRQ | EF, Inh, Up | Modified AB/Delayed Response Task (81^e^) | Obs | | -.11 | 214 |
| Nolvi et al., 2021^f^ | 6.00 | 44 | Com | 100% White/Caucasian | Pos | EPDS (-) | SRQ | SR | IBQ-R-SF ORC (.80) | Q | | -.21 | 93 |
|  | 12.00 |  | Com |  | Pos | EPDS (-) | SRQ | SR | IBQ-R-SF ORC (.80) | Q | | -.04 | 84 |
|  | 12.00 |  | Com |  | Pos | EPDS (-) | SRQ | SR | IBQ-R-SF ORC (.80) | Q | | -.04 | 84 |
| Pacheco & Figueiredo, 2012 | 0.09 | 44 | Com | 100% Caucasian | Pre | EPDS  (.75) | SRQ | State | NBAS State (.54) | CA | | -.20 | 110 |
| Peacock-Chambers, 2016 | 10.40 | 49 | Com | 48% White non-Hispanic, 14% Black non-Hispanic, 20% Hispanic, 18% Other | Pos | CESD (-) | SRQ | SR | ITSC (-) | Q | | -.09 | 5,728 |
| Perez et al., 2022 | 6.50 | 40 | Com | - | Pos | EPDS (.83) | SRQ | Reg | CFS Sleeping/ Crying (.86) | Q | | -.18 | 162 |
| Pinto et al., 2023 | 3.64 | 54 | Com | 91% Portuguese | Pre | EPDS (.80) | SRQ | SR | IBQ-R-SF ORC (.72) | Q | | -.42 | 86 |
|  | 3.64 |  | Com |  | Pos | EPDS (.80) | SRQ | SR | IBQ-R-SF ORC (.72) | Q | | -.29 | 86 |
| Pressman, 2011 | 12.00 | 49 | Com | 45% White, 25% Black, 23% Hispanic, 4% Other | Pos | CESD (.88) | SRQ | ER | BSID Behavior Rating Scale ER (.90) | Obs | | -.07 | 973 |
| Raikkonen et al., 2015 | 0.52 | - | Com | *-* | Pre | CESD (-) | SRQ | Reg | NPI (.71) | Q | | -.18 | 52 |
|  | 0.52 |  | Com |  | Pre | CESD (-) | SRQ | Reg | NPI (.71) | Q | | -.20 | 52 |
|  | 0.52 |  | Com |  | Pre | CESD (-) | SRQ | Reg | NPI (.71) | Q | | -.37 | 52 |
|  | 0.52 |  | Com |  | Pos | CESD (-) | SRQ | Reg | NPI (.71) | Q | | -.27 | 52 |
| Reebye et al., 2012 | 8.00 | 50 | Com | 87% White,  8% Indian/Asian, 3% Other,  2% Native Indian | Pos | HRSD (-) | DI | Mut, SR | SR in Structured Play (-) | Obs | | .16 | 23 |
|  | 8.00 |  | Com |  | Pos | HRSD (-) | DI | Mut | Affective Quality of Interaction in Structured Play (-) | Obs | | -.06 | 23 |
|  | 8.00 |  | Com |  | Pos | HRSD (-) | DI | Mut | Mutuality in Structured Play (-) | Obs | | -.33 | 23 |
|  | 8.00 |  | Com |  | Pos | HRSD (-) | DI | Mut, SR | SR in Free Play (-) | Obs | | .14 | 23 |
|  | 8.00 |  | Com |  | Pos | HRSD (-) | DI | Mut | Affective Quality of Interaction in Free Play (-) | Obs | | -.01 | 23 |
|  | 8.00 |  | Com |  | Pos | HRSD (-) | DI | Mut | Mutuality in Free Play (-) | Obs | | -.06 | 23 |
|  | 8.00 |  | Cl |  | Pos | HRSD (-) | DI | Mut, SR | SR in Structured Play (-) | Obs | | .03 | 16 |
|  | 8.00 |  | Cl |  | Pos | HRSD (-) | DI | Mut | Affective Quality of Interaction in Structured Play (-) | Obs | | .09 | 16 |
|  | 8.00 |  | Cl |  | Pos | HRSD (-) | DI | Mut | Mutuality in Structured Play (-) | Obs | | -.02 | 16 |
|  | 8.00 |  | Cl |  | Pos | HRSD (-) | DI | Mut, SR | SR in Free Play (-) | Obs | | -.21 | 16 |
|  | 8.00 |  | Cl |  | Pos | HRSD (-) | DI | Mut | Affective Quality of Interaction in Free Play (-) | Obs | | -.06 | 16 |
|  | 8.00 |  | Cl |  | Pos | HRSD (-) | DI | Mut | Mutuality in Free Play (-) | Obs | | -.32 | 16 |
|  | 8.00 |  | Cl |  | Pos | HRSD (-) | DI | Mut, SR | SR in Structured Play (-) | Obs | | .35 | 11 |
|  | 8.00 |  | Cl |  | Pos | HRSD (-) | DI | Mut | Affective Quality of Interaction in Structured Play (-) | Obs | | -.34 | 11 |
|  | 8.00 |  | Cl |  | Pos | HRSD (-) | DI | Mut | Mutuality in Structured Play (-) | Obs | | -.09 | 11 |
|  | 8.00 |  | Cl |  | Pos | HRSD (-) | DI | Mut, SR | SR in Free Play (-) | Obs | | .29 | 11 |
|  | 8.00 |  | Cl |  | Pos | HRSD (-) | DI | Mut | Affective Quality of Interaction in Free Play (-) | Obs | | .10 | 11 |
|  | 8.00 |  | Cl |  | Pos | HRSD (-) | DI | Mut | Mutuality in Free Play (-) | Obs | | .16 | 11 |
| Rencken et al., 2022 | 0.07 | 40 | Com | - | Oth | EPDS (-) | SRQ | State | NBAS State (99^g^) | CA | | .15 | 132 |
| Selman et al., 2020 | 12.93  12.93 | - | Com | 62% Latino(a), 14% White, 8% African American, 8% American Indian/Alaskan/ Native, 8% Multiracial | Pre | CESD (.85) | SRQ | EC, ER | IBQ-R-VSF ORC (.81) | Q | | -.37 | 37 |
|  |  |  |  |  | Pos | CESD (.90) | SRQ | EC, ER | IBQ-R-VSF ORC (.81) | Q | | -.48 | 37 |
| Simon, 2020 | 6.44 | - | Com | 81% Caucasian, 8% African American, 3% Asian, 2% Hispanic/Latinx, 7% Other | Pos | DASS Dep (.94) | SRQ | EC | IBQ-R-VSF ORC (.70) | Q | | -.02 | 155 |
| Smith et al., 2012 | 1.00 | 47 | Com | 40% White, 23% Hispanic, 18% Pacific Islander, 12% Asian, 5% Black, 2% American Indian, <1% Other | Pos | BDI-II (-) | SRQ | SR | NNNS  Reg (-) | CA | | -.57 | 314 |
| Thomas et al., 2017 | 6.00 | 48 | Com | 81% White, 6% Asian, 6% Other, 4% Latin American, 3% Chinese | Pre | EPDS  (.82) | SRQ | ER | Attentional Strategies in LabTAB  Frustration (.93^k^) | Obs | | .06 | 254 |
|  | 6.00 |  | Com |  | Pre | EPDS  (.82) | SRQ | ER | Attentional Strategies in LabTAB  Frustration (.93^k^) | Obs | | .00 | 254 |
|  | 6.00 |  | Com |  | Pre | EPDS  (.82) | SRQ | ER | Avoidance Strategies in LabTAB Frustration (.90^k^) | Obs | | .07 | 254 |
|  | 6.00 |  | Com |  | Pre | EPDS  (.82) | SRQ | ER | Avoidance Strategies in LabTAB Frustration (.90^k^) | Obs | | .05 | 254 |
|  | 6.00 |  | Com |  | Pre | EPDS  (.82) | SRQ | ER | Self-Soothing Strategies in LabTAB  Frustration  (.93^k^) | Obs | | -.01 | 254 |
|  | 6.00 |  | Com |  | Pre | EPDS  (.82) | SRQ | ER | Self-Soothing Strategies in LabTAB  Frustration  (.93^k^) | Obs | | -.13 | 254 |
| Thomas & Spieker, 2016 | 8.00 | 45 | Com | 81% White,  10% Asian,  7% Black or African American,  2% Hispanic, 2% Native Hawaiian or Pacific Islander | Pos | PROMIS Dep  (.80) | SRQ | Reg | ITSC (-) | Q | | -.27 | 42 |
| Vaever et al., 2020 | 10.11 | 50 | Cl | - | Pos | PSE (-) | DI | SR | High NA Duration in Mother-Infant Interaction (.60^h^) | Obs | | .14 | 76 |
|  | 10.11 |  | Cl |  | Pos | PSE (-) | DI | SR | High NA Frequency in Mother-Infant Interaction (.60^h^) | Obs | | .22 | 76 |
|  | 10.11 |  | Cl |  | Pos | PSE (-) | DI | SR | Gaze Off Duration in Mother-Infant Interaction (.60^h^) | Obs | | .05 | 71 |
|  | 10.11 |  | Cl |  | Pos | PSE (-) | DI | SR | Gaze Off Frequency in Mother-Infant Interaction (.60^h^) | Obs | | -.26 | 71 |
| van Huisstede, 2019^i^ | 6.00 | 54 | Com | 100% Mexican-American | Pos | EPDS (.83) | SRQ | SR | Dysreg during soothing task (-) | Obs | | -.07 | 194 |
|  | 6.00 |  | Com |  | Pos | EPDS (.83) | SRQ | SR | Dysreg during teaching task (-) | Obs | | -.03 | 194 |
|  | 6.00 |  | Com |  | Pos | EPDS (.83) | SRQ | SR | Dysreg during peek-a-boo task (-) | Obs | | -.14 | 194 |
| Vieites & Reeb-Sutherland, 2017 | *-* | 50 | Com | 50% Caucasian, 20% African American,  3% Asian,  3% Hispanic,  2% Mixed | Pos | BDI (.73) | SRQ | ER | Object/ Environment Engagement and Oral Self-Comforting in SFP (-) | Obs | | .23 | 63 |
| Villani et al., 2018 | 15.41 | 52 | Com | 72% Caucasian, 10% Asian, 4% African Canadian, 14% Other Ethnicities | Pos | BDI-II (.89) | SRQ | ER, SR | Independent ER in Toy Frustration (.79^k^) | Obs | | .17 | 172 |
| Woolard et al., 2023 | 12.00 | 43 | Com | - | Pos | EPDS (-) | SRQ | Reg | FYI Sens (-) | Q | | -.20 | 78 |
| Wu & Gazelle, 2021 | 6.00 | 49 | Com | 59% Caucasian, 41% African American, <1% Other Races | Pos | BSI-18 Dep (.81) | SRQ | Fear | Avoidance during Mask Task (.82^h^) | Obs | | -.02 | 1,057 |
|  | 15.00 |  | Com |  | Pos | BSI-18 Dep (.81) | SRQ | Fear | Avoidance during Mask Task (.82^h^) | Obs | | -.01 | 892 |
|  | 15.00 |  | Com |  | Pos | BSI-18 Dep (.83) | SRQ | Fear | Avoidance during Mask Task (.82^h^) | Obs | | .01 | 909 |
|  | 6.00 |  | Com |  | Pos | BSI-18 Dep (.81) | SRQ | Fear | OtM during Mask Task (.82^h^) | Obs | | -.01 | 1,057 |
|  | 15.00 |  | Com |  | Pos | BSI-18 Dep (.81) | SRQ | Fear | OtM during Mask Task (.82^h^) | Obs | | -.04 | 892 |
|  | 15.00 |  | Com |  | Pos | BSI-18 Dep (.83) | SRQ | Fear | OtM during Mask Task (.82^h^) | Obs | | -.04 | 909 |
|  | 6.00 |  | Com |  | Pos | BSI-18 Dep (.81) | SRQ | Fear,AR | AR during Mask Task (.82^h^) | Obs | | .01 | 1,057 |
|  | 15.00 |  | Com |  | Pos | BSI-18 Dep (.81) | SRQ | Fear,AR | AR during Mask Task (.82^h^) | Obs | | .02 | 892 |
|  | 15.00 |  | Com |  | Pos | BSI-18 Dep (.83) | SRQ | Fear,AR | AR during Mask Task (.82^h^) | Obs | | .02 | 909 |
|  | 6.00 |  | Com |  | Pos | BSI-18 Dep (.81) | SRQ | Fear | Self-sooth during Mask Task (.82^h^) | Obs | | -.03 | 1,057 |
|  | 15.00 |  | Com |  | Pos | BSI-18 Dep (.81) | SRQ | Fear | Self-sooth during Mask Task (.82^h^) | Obs | | .01 | 892 |
|  | 15.00 |  | Com |  | Pos | BSI-18 Dep (.83) | SRQ | Fear | Self-sooth during Mask Task (.82^h^) | Obs | | -.05 | 909 |

*Note.* Dashes indicate that data were not available. Reliability was reported in Cronbach’s alpha unless otherwise noted. Age is infant age in months. Sex is infant sex in percent female.

AC = Attentional control; AR = Attention regulation; AR = Arm Restraint task**;** ASQ-SE = Ages and Stages Questionnaire-Social Emotional; BDI(-II) = Beck Depression Inventory(-II); BR = Behavioral regulation; BSID = Bayley Scales of Infant Development; BSI-18 Dep = Brief Symptom Inventory-18 Depressive Symptoms subscale; CA = Clinician assessment; CESD = Center for Epidemiological Studies Depression scale; CFS = Questionnaire for Crying, Feeding, and Sleeping; CIDI = Composite International Diagnostic Interview; Cl = Clinical; Com = Community; Con = Self-regulation-related construct; CPQ = Crying Patterns Questionnaire; DASS Dep = Depression and Anxiety Stress Scale Depression subscale; DI = Diagnostic interview; DIS = Diagnostic Interview Schedule; DL = Distress to Limitations subscale**;** DO = Duration of Orienting subscale**,** DVR = Decreased Vocal Reactivity subscale**;** Dyadic Reg = Dyadic regulation; Dysreg = Dysregulation scale; EC = Effortful control; EF = Executive functioning; EPDS = Edinburgh Postnatal Depression Scale; ER = Emotion regulation; ET = Eye tracking; Fear = Fear regulation; FPS = Feeding Problems Scale; FR = Falling Reactivity subscale; FYI Sens = Sensory Regulation domain of the First Year Inventory**;** HRSD = Hamilton Rating Scales for Depression; IBQ(-R-[V]SF) = Infant Behavior Questionnaire(-Revised-[Very] Short Form); Inh = Inhibition; Int = Interview; ITSC = Infant Toddler Symptom Checklist; ITSEA = Infant and Toddler Social-Emotional Assessment; LabTAB = Laboratory Temperament Assessment Battery; LIP = Low-Intensity Pleasure subscale**,** LTH = Lifetime history**;** MADR = Montgomery-Asberg Depression Rating scale; MC = Extracted from medical chart; Met = Method**;** MINI = Mini International Neuropsychiatric Interview; Mut = Mutual regulation; NA = Negative affectivity; NBAS = Neonatal Behavioral Assessment Scale; NE = Negative Emotionality subscale; NM = Negative mood; NPI = Neonatal Perception Inventory; Neur = Neurobehavioral regulation; NNNS = NICU Network Neurobehavioral Scale; Obs = Observational coding of task; ORC = Orienting/Regulatory Control scale; Oth = Other**;** OtM = Orienting to mother; PDSS = Postpartum Depression Screening Scale; Pos = Postnatal**;** Pre = Pregnancy; PR = Positive recovery; PROMIS Dep = Patient Reported Outcomes Measurement Information System-Depression scale; PSE = Present State Examination; Q = Questionnaire; Reg = Regulation; RPQ = Regulatory Problems Questionnaire; Sam = Sample type**;** SCID-IV(-R) = Structured Clinical Interview for DSM-IV(-Revised); SCL-90-R Dep = Symptom Checklist-90-Revised Depression scale; SFP = Still Face Paradigm**;** SHS = Sleep Habits Scale; Sooth = Soothability subscale; SR = Self-regulation; SRQ = Self-report questionnaire; State = State regulation; Str = Stress regulation; Tim = Maternal depression timing; TS = Tactile Sensitivity subscale; Up = Updating; ZSRD = Zung Self-Rating Depression Scale.

^a^ Pearson product-moment correlation coefficients. All effect sizes were coded so that negative associations indicated that higher levels of maternal depression were associated with worse infant self-regulation.

^b^ Only Spearman’s correlations were provided and are included in the table.

^c^ Infant race, used only when maternal race was not reported.

^d^ The analyses presented in these manuscripts were identified as including the same samples; thus, the effect sizes drawn from these manuscripts were treated as nested within the same study in analyses.

^e^ Reliability was McDonald’s Omega.

^f^ The analyses presented in these manuscripts were identified as including the same samples; thus, the effect sizes drawn from these manuscripts were treated as nested within the same study in analyses.

^g^ Reliability was percent interrater agreement.

^h^ Reliability was Cohen’s kappa.

^i^ The analyses presented in these manuscripts were identified as including the same samples; thus, the effect sizes drawn from these manuscripts were treated as nested within the same study in analyses.

^j^ The analyses presented in these manuscripts were identified as including the same samples; thus, the effect sizes drawn from these manuscripts were treated as nested within the same study in analyses.

^k^ Reliability was intraclass correlation coefficient.
